# Mitochondrial Glycolysis in a Major Lineage of Eukaryotes

**DOI:** 10.1093/gbe/evy164

**Published:** 2018-07-30

**Authors:** Carolina Río Bártulos, Matthew B Rogers, Tom A Williams, Eleni Gentekaki, Henner Brinkmann, Rüdiger Cerff, Marie-Françoise Liaud, Adrian B Hehl, Nigel R Yarlett, Ansgar Gruber, Peter G Kroth, Mark van der Giezen

**Affiliations:** 1 Institut für Genetik, Technische Universität Braunschweig; 2 Fachbereich Biologie, Universität Konstanz, Germany; 3 Biosciences, University of Exeter, United Kingdom; 4 School of Biological Sciences, University of Bristol, United Kingdom; 5 Department of Biochemistry & Molecular Biology, Dalhousie University, Halifax, Canada; 6 Département de Biochimie, Université de Montréal C.P. 6128, Montréal, Quebec, Canada; 7 Institute of Parasitology, University of Zürich, Switzerland; 8 Department of Chemistry and Physical Sciences, Pace University; 9 Rangos Research Center, University of Pittsburgh, Children's Hospital, Pittsburgh, PA; 10 School of Science and Human Gut Microbiome for Health Research Unit, Mae Fah Luang University, Chiang Rai, Thailand; 11 Leibniz-Institut DSMZ—Deutsche Sammlung von Mikroorganismen und Zellkulturen GmbH, Braunschweig, Germany; 12 Institute of Parasitology, Biology Centre, Czech Academy of Sciences, České Budějovice, Czech Republic

**Keywords:** glycolysis, mitochondria, organelle, stramenopile, evolution, compartmentalization

## Abstract

The establishment of the mitochondrion is seen as a transformational step in the origin of eukaryotes. With the mitochondrion came bioenergetic freedom to explore novel evolutionary space leading to the eukaryotic radiation known today. The tight integration of the bacterial endosymbiont with its archaeal host was accompanied by a massive endosymbiotic gene transfer resulting in a small mitochondrial genome which is just a ghost of the original incoming bacterial genome. This endosymbiotic gene transfer resulted in the loss of many genes, both from the bacterial symbiont as well the archaeal host. Loss of genes encoding redundant functions resulted in a replacement of the bulk of the host’s metabolism for those originating from the endosymbiont. Glycolysis is one such metabolic pathway in which the original archaeal enzymes have been replaced by bacterial enzymes from the endosymbiont. Glycolysis is a major catabolic pathway that provides cellular energy from the breakdown of glucose. The glycolytic pathway of eukaryotes appears to be bacterial in origin, and in well-studied model eukaryotes it takes place in the cytosol. In contrast, here we demonstrate that the latter stages of glycolysis take place in the mitochondria of stramenopiles, a diverse and ecologically important lineage of eukaryotes. Although our work is based on a limited sample of stramenopiles, it leaves open the possibility that the mitochondrial targeting of glycolytic enzymes in stramenopiles might represent the ancestral state for eukaryotes.

## Introduction

Mitochondria provide the bulk of cellular ATP for eukaryotes via oxidative phosphorylation, also known as cellular respiration (Müller et al. 2012). In addition, mitochondria are essential for the production of iron–sulfur clusters (Lill et al. 1999) and play roles in heme synthesis, in fatty acid and in amino acid metabolism (Scheffler 2008). For cellular respiration, pyruvate is produced in the cytosol via glycolysis and imported into the mitochondrion. The pyruvate is then decarboxylated by a mitochondrial pyruvate dehydrogenase to acetyl-CoA. This acetyl-CoA enters the citric acid cycle, subsequently producing one GTP (or ATP) and precursors for several anabolic pathways. More importantly, the reduction of NAD^+^ to NADH and the production of succinate power the respiratory electron transport chain and subsequently ATP synthesis by the proton gradient driven ATP synthase, which is responsible for the majority of cellular ATP synthesis.

Glycolysis, the pathway that produces the pyruvate, is a widespread metabolic pathway that converts the six-carbon sugar glucose via a series of ten reactions into the three-carbon sugar pyruvate. During this conversion, energy is stored (two ATP per glucose) and reducing equivalents are formed (two NADH per glucose). To keep the pathway going, NADH needs to be recycled to NAD^+^, which can happen in a fermentative process, most commonly leading to the formation of lactic acid or ethanol in the cytosol, or by shuttling of the reducing equivalents to the mitochondrial respiratory electron transport chain, which leads to an increased ATP yield.

Glycolysis is present in all known eukaryotes, with the exception of some extremely reduced intracellular parasites (Keeling et al. 2010; Wiredu Boakye et al. 2017). Glycolysis is nearly universally present in the cytosol of most eukaryotes and also found in specialized microbodies known as glycosomes, originally found in trypanosomes (Opperdoes and Borst 1977), but more recently found to be perhaps a more general feature of all the euglenozoa (Morales et al. 2016). Two glycolytic enzymes were also found to be targeted to peroxisomes in fungi due to posttranscriptional processes (Freitag et al. 2012). An unusual TPI-GAPDH fusion protein was reported to localise to the mitochondrion of a stramenopile, the diatom *Phaeodactylum tricornutum* (Liaud et al. 2000). In addition, bioinformatics studies (Kroth et al. 2008; Nakayama et al. 2012) have hinted at the possible mitochondrial location of several glycolytic enzymes.

Stramenopiles are a large and extremely diverse eukaryotic group of organisms, including phototrophic members such as the multicellular kelps (brown algae) and unicellular microalgae including diatoms. They also include nonphototrophic members such as oomycetes, plant pathogens with an enormous impact on agriculture (Jiang and Tyler 2012) and the human pathogen *Blastocystis* (Stensvold and van der Giezen 2018). The latter species has an anaerobic lifestyle and lacks many features commonly found in mitochondria (Müller et al. 2012; Gentekaki et al. 2017). The stramenopiles evolved by endosymbiotic uptake of a red alga but the question as to whether the nonphotosynthetic members never possessed a plastid, or simply lost it, remains unclear (Baurain et al. 2010; Petersen et al. 2014; Derelle et al. 2016). Members of the stramenopiles can be found in most ecosystems on earth: in marine and fresh water environments, in soil, and as pathogens of humans, animal and plants. Despite their enormous variety in lifestyle a clear monophyly of this group is undisputed (Walker et al. 2011; Derelle et al. 2016).

Here, we report that the second half of glycolysis, the C3 part, is targeted to mitochondria in the stramenopiles. This exclusive feature of the stramenopiles might be a synapomorphy of this large group of eukaryotes. Mitochondrial glycolysis only covers the pay-off phase of glycolysis, in which the three carbon sugars are converted to pyruvate, leading to the release of energy and reducing equivalents in the form of ATP and NADH.

## Materials and Methods

### Sources of cDNA and Genomic DNA

DNA and cDNA from *Blastocystis* ST1 strain NandII, obtained from a symptomatic human (strain obtained from the American Type Culture Collection, ATCC 50177), was used in this study. Genomic and cDNA libraries of *Phaeodactylum tricornutum* (culture from SAG strain: 1090-1a, Göttingen) were constructed with the “Lambda ZAP II XR library Construction Kit” from Stratagene and the lambda vector EMBL3, respectively. *P. tricornutum* Bohlin (strain 646; University of Texas Culture Collection, Austin) RNA was isolated using TRIzol following manufacture’s protocol (Thermo Fisher, Germany) and cDNA synthesis was performed with the reverse Transcription system (A3500, Promega, Germany). An *Achlya bisexualis* cDNA library (Bhattacharya et al. 1991) was kindly provided by D. Bhattacharya (Rutgers University). Screening of libraries, sequencing of positive clones and RACE analyses were performed as described (Liaud et al. 1997). *Phytophthora infestans* RNA extracted from *P. infestans* mycelia with the RNAeasy Plant Kit from Quiagen and cDNA was synthesized with the Thermo-RT Kit (Display Systems, England). Sequences were also obtained from the EST/genome sequencing programmes from *Phaeodactylum tricornutum* (Maheswari et al. 2005) and http://genome.jgi-psf.org/Phatr2/Phatr2.home.html (JGI)(Bowler et al. 2008), from *Phytophthora infestans* ([Tripathy et al. 2006]) and from *Phytophtora sojae* and *Phytophtora ramorum* (http://www.jgi.doe.gov [Tyler et al. 2006]).

### GFP Constructs for the Stable Transformation of *Phaeodactylum tricornutum*

Standard cloning procedures were applied (Sambrook et al. 1989). Polymerase chain reaction (PCR) was performed with a Master Cycler Gradient (Eppendorf) using Taq DNA Polymerase (Q BIOgene) according to the manufacturer’s instructions. cDNA from *Blastocystis* ST1 strain NandII (Bl), *Phaeodactylum tricornutum* (Pt), *Phytophthora infestans* (Pi) and *Achlya bisexualis* (Ab) was used as template for the PCR reactions. For *Saccharina latissima* (Sl) a cDNA clone (NCBI: ABU96661) was used as template. PCR products were cloned into TA-vector PCR 2.1 (Invitrogen) or blunt cloned into pBluescript II SK+ (Stratagene). The primers (supplementary table 1, Supplementary Material online) allowed insertion of restriction enzyme recognition sites (*Eco*RI/*Nco*I or *Sma*I/*Nco*I) that were used to clone the presequences in frame to eGFP within pBluescript-GFP. The presequence-GFP fusions were cut out with appropriate restrictions enzymes (*Eco*RI/*Hin*dIII or *Sma*I/*Hin*dIII) and cloned into the *Phaeodactylum tricornutum* transformation vector pPha-T1 (Zaslavskaia et al. 2000; Gruber et al. 2007), either into the corresponding sites or, in case of *Sma*I, into the *Eco*RV site. For the constructs with Protein ID (fig. 5) a slightly different cloning approach was used. PCR with a proof reading Polymerase (*Pfu* or Kapa Hifi) were used to amplify corresponding fragments from cDNA. The fragments were cloned blunt end in a modified pPha-T1 Vector. The vectors include an eGFP with a *Stu*I or *Ksp*AI restriction site, allowing a one-step cloning procedure, with subsequent screening for the correct orientation of the fragment at the N-terminus of eGFP. The *Blastocystis* presequences were produced by kinasing the primers using T4 polynucleotide kinase using manufacturer’s procedures and subsequently annealing in a thermal cycler after which they were cloned into the diatom expression vector equipped with eGFP and the *Stu*I restriction site.

### Cultivation and Transformation of *Phaeodactylum tricornutum*

*Phaeodactylum tricornutum* Bohlin (UTEX, strain 646) was grown at 22 °C under continuously light of 75 µE in artificial seawater (Tropic Marin) at a 0.5 concentration. Transformations were performed as described by Zaslavskaia et al. (Zaslavskaia et al. 2000; Kroth 2007). For each transformation, tungsten particles M10 (0.7 μm median diameter) covered with 7–20 μg DNA were used to bombard cells with the Particle Delivery System PDS-1000 (Bio-Rad, HE-System) prepared with 650, 900, 1100, or 1350 psi rupture discs.

### Microscopic Analysis of Transformed *Phaeodactylum tricornutum*

Reporter gene expression was visualized using confocal laser scanning microscopy (cLSM-510META, Carl Zeiss, Jena, Germany) using a Plan-Neofluar 40×/1.3 Oil DIC objective. The eGFP fusion proteins were excited with an argon laser at 488 nm with 8–10% of laser capacity. Excited fluorophores were detected with a bandpass filter GFP (505–530 nm) using a photomultiplier. Chlorophyll *a* autofluorescence was simultaneously detected with a META-channel (644–719 nm). MitoTraker Orange CM-H_2_TMRos (Molecular Probes) was applied for fluorescence staining of mitochondria. *P. tricornutum* cells were stained according to the protocol of the manufacturer. Cells were incubated with 100 nM dye solution, incubated for 30 min, washed and observed (images were recorded using the Multitracking mode with the following parameters for Wavelength T1 = 488 nm 10% and T2 = 543 nm 100% laser line, primary beam splitting mirrors UV/488/543/633 nm; emitted light was detected with the META-channel).

### Protein Production and Antibody Generation

*Blastocystis* TPI-GAPDH was amplified from cDNA using primers TPI-GAPDH pET F: aga aga *CAT ATG* TTC GTC GGT GGC AAT TGG AAG TGC AA and TPI-GAPDH pET R: tct tct *GGA TCC* TTA AGA GCG ATC CAC CTT CGC CA adding *Nde*I and *Bam*HI restriction sites, respectively, to facilitate cloning in gene expression vector pET14b (Novagen, Merck, Whatford, UK). The *Blastocystis* PGK was amplified from cDNA using PGK pET F: aga aga *CAT ATG* AAG CTG GGA GTT GCT GCC TAC G and PGK pET R: tct tct *CAT ATG* TCA CGC GTC CGT CAG AGC GGC CAC ACC C which added *Nde*I restriction sites for pET14b cloning. The mitochondrial targeting signals were not amplified as these would not be part of the mature processed protein. All constructs were confirmed by sequencing. The in-frame His-tag allowed for affinity chromatography purification of the recombinant protein. Recombinant *Blastocystis* TPI-GAPDH and PGK were used to immunise guinea pigs and rabbits, respectively, for polyclonal antibody generation at Eurogentec (Seraing, Belgium).

### Culture Conditions for *Blastocystis*

*Blastocystis* isolate B (originally designated *Blastocystis* sp. group VII [Noel et al. 2005], now called ST7 [Stensvold et al. 2007]) was used. The parasite was grown in 10 ml pre-reduced Iscove’s modified Dulbecco’s medium (IMDM) supplemented with 10% heat-inactivated horse serum. Cultures were incubated for 48 h in anaerobic jars using an Oxoid AneroGen pack at 37 °C. Two-day-old cultures were centrifuged at 1,600 × g for 10 min, washed once in a buffer consisting of 30 mM potassium phosphate, 74 mM NaCl, 0.6 mM CaCl_2_ and 1.6 mM KCl, pH 7.4 and resuspended in a nitrogen gassed isotonic buffer consisting of 200 mM sucrose (pH 7.2) containing 30 mM phosphate, 15 mM mercaptoethanol, 30 mM NaCl, 0.6 mM CaCl_2_, and 0.6 mM KCl (pH 7.2).

### Confocal Microscopy of *Blastocystis*

*Blastocystis* trophozoites were treated with MitoTracker Red (Molecular Probes), washed, fixed in 10% formalin and incubated in ice cold acetone for 15 min and air-dried.

Slides with fixed parasites were rehydrated in phosphate buffered saline (PBS) for 30 min and blocked with 2% BSA in PBS for 1 hr at room temperature. All antibody incubations were performed at room temperature in 2% BSA in PBS, 0.1% triton X-100. Slides were washed five times in 0.2% BSA in PBS, 0.01% triton X-100 between incubations to remove unbound antibodies.

Primary antibodies: Rabbit, anti-PGK; Guinea Pig, anti-TPI-GAPDH (Eurogentec, Seraing, Belgium) were used at a dilution of 1:500 and 1:300 in 2% BSA in PBS, 0.1% triton X-100, respectively.

Secondary antibodies: Alexa Fluor 488 conjugated Goat anti-Rabbit (Invitrogen, Eugene, OR, USA), Alexa Fluor 405 conjugated Goat anti-Rabbit (Invitrogen, Eugene, OR, USA), TRITC-conjugated Goat anti-Guinea Pig were used at 1:200 dilutions in 2% BSA in PBS, 0.1% triton X-100, each.

The DNA intercalating agent 4'-6-Diamidino-2-phenylindole (DAPI) for detection of nuclear and mitochondrial DNA was added to the final but one washing solution at a concentration of 1 µg·ml^−1^. The labeled samples were embedded in Dako Glycergel Mounting Medium (DAKO, Carpinteira, CA, USA) and stored at 4 °C.

Immunofluorescence analysis and image data collection was performed on a Leica SP2 AOBS confocal laser-scanning microscope (Leica Microsystems, Wetzlar, Germany) using a glycerol immersion objective lens (Leica, HCX PL APO CS 63x 1.3 Corr). Image z-stacks were collected with a pinhole setting of Airy 1 and twofold oversampling. Image stacks of optical sections were further processed using the Huygens deconvolution software package version 2.7 (Scientific Volume Imaging, Hilversum, NL). Three-dimensional reconstruction, volume and surface rendering, and quantification of signal overlap in the 3D volume model were generated with the Imaris software suite (Version 7.2.1, Bitplane, Zurich, Switzerland). The degree of signal overlap in the 3D volume model is depicted graphically as scatterplots. The intensity of two fluorescent signals in each voxel of the 3D model is measured and plotted. Voxels with similar signal intensity for both signals appear in the area of the diagonal. All image stacks were corrected for spectral shift before rendering and signal colocalization analysis.

### Subcellular Fractionation of *Blastocystis*

*Blastocystis* cells were broken by mixing two volumes of the cell suspension with three volumes of 0.5 mm beads and broken by three 1 min duration shakes at maximum speed on a bead breaker (VWR mini bead mill homogenizer, Atlanta, GA, USA) with 1-min pauses on ice. Cell-free extracts were subjected to increasing centrifugal force producing nuclear (N, 1,912 RCF_av_ for 5 min), mitochondria-like (ML, 6,723 RCF_av_ for 15 min), lysosomal (L, 26,892 RCF_av_ for 30 min) and cytosolic (S) fractions, respectively, using a using a Sorvall RC-2B centrifuge fitted with an SS-34 rotor.

### Enzyme Assays

Hexokinase was assayed by measuring the reduction of NAD^+^ at 340 nm in a coupled reaction with *Leuconostoc mesenteroides* glucose-6-phophate dehydrogenase (3 EU), containing 38 mM Tris–HCl pH 7.6, 115 mM D-glucose, 10 mM MgCl_2_, 0.5 mM ATP, 0.2 mM NAD^+^, 0.05 ml of *Blastocystis* cell-free extract (0.08–0.12 mg) or fraction (N, 0.15–0.18 mg; ML, 0.12–0.17 mg; L, 0.08–0.11 mg; S, 0.09–0.05 mg), in a final volume of 1 ml at 25 °C.

Phosphoglucose isomerase was assayed by measuring the reduction of NADP^+^ at 340 nm in a coupled reaction with *Leuconostoc mesenteroides* glucose-6-phophate dehydrogenase (2 EU), containing 38 mM Tris–HCl pH 7.6, 3.3 mM D-fructose-6-phosphate, 0.66 mM β-NADP^+^, 3.3 mM MgCl_2_, 0.05 ml of *Blastocystis* cell-free extract or fraction in a final volume of 3 ml at 25 °C.

Phosphofructokinase was assayed using the standard coupled assay containing 38 mM Tris–HCl pH 7.6, 5 mM dithiothreitol, 5 mM MgCl_2_, 0.28 mM NADH, 0.1 mM ATP, 0.1 mM AMP, 0.8 mM fructose-6-ohosphate, 0.4 mM (NH_4_)_2_SO_4_, 0.05 EU each of rabbit muscle aldolase, rabbit muscle glycerophosphate dehydrogenase, and rabbit muscle triosephosphate isomerase, 0.05 ml of *Blastocystis* cell-free extract or fraction in a final volume of 3 ml at 25 °C.

Aldolase was assayed using a modification of the hydrazine method in which 3-phosphoglyceraldehyde reacts with hydrazine to form a hydrazone which absorbs at 240 nm; the assay contained 12 mM fructose-1,6-bisphosphate, pH 7.6, 0.1 mM EDTA, 3.5 mM hydrazine sulfate and 0.05 ml of *Blastocystis* cell-free extract or fraction in a final volume of 3 ml at 25 °C.

Triosephosphate isomerase was assayed by measuring the oxidation of NADH using a linked reaction with glycerol-3-phosphate dehydrogenase; 220 mM triethanolamine pH 7.6, 0.20 mM DL-glyceraldehyde-3-phosphate, 0.27 mM NADH, 1.7 EU glycerol-3-phosphate dehydrogenase, and 0.05 ml of *Blastocystis* cell-free extract or fraction in a final volume of 3 ml at 25 °C.

Glyceraldehyde-3-phosphate dehydrogenase was assayed by measuring the initial reduction of NAD^+^ at 340 nm; the assay contained 13 mM sodium pyrophosphate pH 8.0, 26 mM sodium arsenate, 0.25 mM NAD, 3.3 mM dithiothreitol, and 0.05 ml of *Blastocystis* cell-free extract or fraction in a final volume of 3 ml at 25 °C.

Phosphoglycerate kinase was assayed by measuring the 3-phosphoglycerate dependent oxidation of NADH at 340 nm; the assay contained 40 mM Tris–HCl pH 8.0, 0.5 mM MgCl_2_, 0.26 mM NADH, 0.1 mM ATP, 2 EU *S. cerevisiae* glyceraldehydephosphate dehydrogenase, and 0.05 ml of *B. hominis* cell free extract or fraction in a final volume of 3 ml at 25 °C.

Phosphoglycerate mutase was measured using the standard coupled assay and measuring the decrease in absorbance at 340 nm; the assay contained 76 mM triethanolamine pH 8.0, 7 mM D(-) 3-phosphoglyceric acid, 0.7 mM ADP, 1.4 mM 2,3-diphosphoglyceric acid, 0.16 mM NADH, 2.6 mM MgSO_4_, 100 mM KCl, 5 EU pyruvate kinase/8 EU lactate dehydrogenase from rabbit muscle, 5 EU rabbit muscle enolase, and 0.05 ml of *Blastocystis* cell-free extract or fraction in a final volume of 3 ml at 25 °C.

Enolase was determined using the standard coupled assay and measuring the decrease in absorbance at 340 nm; the assay contained 80 mM triethanolamine pH 8.0, 1.8 mM D(+) 2-phospholycerate, 0.1 mM NADH, 25 mM MgSO_4_, 100 mM KCl, 1.3 mM ADP, 5 EU pyruvate kinase/8 EU lactate dehydrogenase from rabbit muscle, and 0.05 ml of *Blastocystis* cell-free extract or fraction in a final volume of 3 ml at 25 °C.

Pyruvate kinase was determined by measuring the oxidation of NADH at 340 nm using the following mixture, 45 mM imidazole-HCl pH 8.0, 1.5 mM ADP, 0.2 mM NADH, 1.5 mM phosphoenolpyruvate, 5 EU rabbit muscle lactate dehydrogenase, and 0.05 ml of *Blastocystis* cell-free extract or fraction in a final volume of 3 ml at 25 °C.

Pyruvate phosphate dikinase was assayed spectrophotometrically by measuring the oxidation of NADH at 340 nm in 3 ml cuvettes. The reaction contained HEPES buffer (pH 8.0), 6 mM MgSO_4_, 25 mM NH_4_Cl, 5 mM dithiothreitol, 0.1 mM disodium pyrophosphate, 0.25 mM AMP, 0.1 mM phosphoenolpyruvate, and 0.05–0.25 mg of *Blastocystis* cell-free extract or fraction. The rate of pyruvate production was determined by the addition of 2 U of lactate dehydrogenase and 0.25 mM NADH, and compared with controls with phosphoenolpyruvate but lacking AMP, and those containing AMP but lacking phosphoenolpyruvate. The concentration of AMP, pyrophosphate and phosphoenolpyruvate used in the assay was selected from preliminary assays using varying concentrations from 0.025 to 1.0 mM. The generation of ATP from AMP by pyruvate phosphate dikinase was confirmed by measuring the ATP formed using a luciferin/luciferse assay (Molecular Probes, In Vitrogen, Eugene, OR, USA). The assay was performed as described above but lacking lactate dehydrogenase and NADH, after varying times 0, 15, 30, 45, and 60 min 0.1 ml of the assay is removed and added to one well of a 96 well plate containing 0.1 ml of 0.25 μg firefly luciferase and 0.5 mM luciferin and the luminescence recorded using a Spectra Max M2 plate reader (Molecular Devices, Sunnyvale, CA).

The activity of pyrophosphate dependent phosphofructokinase* in the direction of fructose-1,6-bisphosphate formation (forward reaction) was determined in 1 ml assay volumes containing 0.1 M HEPES-HCl, pH 7.8; 20 mM fructose-6-phosphate; 2 mM Na pyrophosphate; 5 mM MgCl_2_; 0.25 mM NADH; 0.2 U of aldolase (from rabbit muscle); and 0.3 U each of glycerophosphate dehydrogenase (from rabbit muscle) and triosephosphate isomerase (from rabbit muscle), 10 μM fructose 2,6 diphosphate. The reaction was initiated by addition of 0.05–0.25 mg of *Blastocystis* cell-free extract or fraction, and the rate of NADH oxidation was followed at 340 nm on a Beckman DU 640 spectrophotometer (Indianapolis, IN, USA). The activity of the reverse reaction was determined by measuring orthophosphate-dependent formation of fructose-6-phosphate from fructose-1,6-bisphosphate. The reaction mixture (1 ml) contained 0.1 M HEPES-HCl, pH 7.8; 2 mM fructose-1,6-bisphosphate; 15 mM NaH2PO4; 5 mM MgCl2; 0.3 mM NADP^+^ and 0.12 U glucose-6-phosphate dehydrogenase and 0.24 U glucose phosphate isomerase. The reaction was initiated by addition of 1 mg of pyrophosphate dependent phosphofructokinase and monitored at 340 nm. *Pyrophosphate fructose-6-phosphate 1-phosphotransferase (PFP).

### Phylogenetic Analyses

Sequences were automatically added to preexisting alignments and subsequently manually refined using the Edit option of the MUST package (Philippe 1993). Final datasets were generated after elimination of highly variable regions and positions with >50% gaps by G-blocks (Talavera and Castresana 2007). All datasets were first analyzed with a maximum likelihood (ML) method under two different models. PhyML v2.3 (Guindon and Gascuel 2003) was used with the SPR moves option and the LG+F + 4G model (Le et al. 2008) and PhyML v3 (with SPR moves) was used using the C20 + 4G model, corresponding to 20 precalculated fixed profiles of positional amino-acid substitution (Le et al. 2008). Based on the likelihood values (*l*), the number of parameters (*K*) and alignment positions (*n*), the AIC (AIC= −2 *l* + 2*K*) and the corrected AIC (AICc; AIC+ 2*K*(*K* + 1)/*n* − *K* − 1) was calculated (Posada and Buckley 2004). The lowest AICc value corresponds to the best tree, if the value of the C20 analysis was better, then a second ML analysis under the C40 + 4G model was performed and the AICc value estimated, until the overall best model was found. If the AICc of C40 is better than C20 then C60 was tested. Once the best model was estimated for all six datasets, a rapid bootstrap analysis with 100 replicates in RAxML v7 under the LG model was performed (Stamatakis et al. 2008) and an additional analysis in Phylobayes v3 with the CATfix C20 model in all cases or, alternatively, the best C-model. Two independent chains were run for 10,000 points and trees are sampled at every tenth points (Lartillot et al. 2009). Trees obtained with the best model are presented and both posterior probabilities (PP) and rapid bootstrap values (BS) are indicated on trees if PP > 0.5 or BS >30%, respectively.

### Cellular Localization Predictions

TargetP (Emanuelsson et al. 2007) and MitoProt (Claros and Vincens 1996) were used to analyse putative subcellular localization. Nonplant and no cut-offs settings were used. However, in case of Viridiplantae, Rhodophyta, and Glaucocystophyta, the plant settings were used if non-plant results differ. Because stramenopiles plastids arose via secondary endosymbiosis, their plastids are contained within the ER and proteins destined for the plastid contain an initial signal peptide. This generally results in erroneous predictions if plant settings are used (Gruber and Kroth 2014, 2017).

## Results

**Fig. 1.**
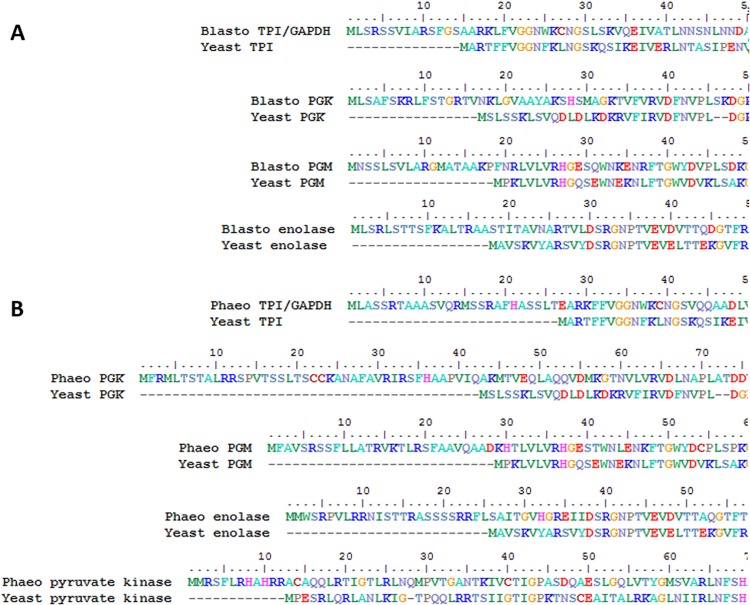
—Stramenopile glycolytic C3 enzymes contain amino-terminal targeting signals. (*A*) Comparison of *Blastocystis* amino-terminal sequences for TPI-GAPDH, PGK, PMG, and enolase with homologs from yeast showing the mitochondrial-like targeting signals. (*B*) *Phaeodactylum tricornutum* glycolytic C3 enzyme amino-termini of TPI-GAPDH, PGK, PMG, enolase, and pyruvate kinase compared with yeast homologs demonstrate mitochondrial-like targeting signals.

When assembling the genome of the intestinal parasite *Blastocystis* (Gentekaki et al. 2017), we discovered putative mitochondrial targeting signals on phosphoglycerate kinase (PGK) as well as on a fusion protein of triose phosphate isomerase (TPI) and glyceraldehyde phosphate dehydrogenase (GAPDH). The amino-terminal sequences conform to typical mitochondrial targeting signals and are enriched in alanine, leucine, serine and arginine (Neupert and Herrmann 2007) and are easily predicted by programmes such as MitoProt (Claros and Vincens 1996). Analyses of the *Blastocystis* TPI-GAPDH and PGK sequences predict mitochondrial localization of these proteins with high probabilities (*P* value 0.99 and 0.97, respectively). The predicted cleavage sites coincide with the start of the cytosolic enzymes from other organisms (fig. 1*A*) suggesting that these amino-terminal sequences might target both proteins to the unusual mitochondrial organelle in this parasite.

**Fig. 2.**
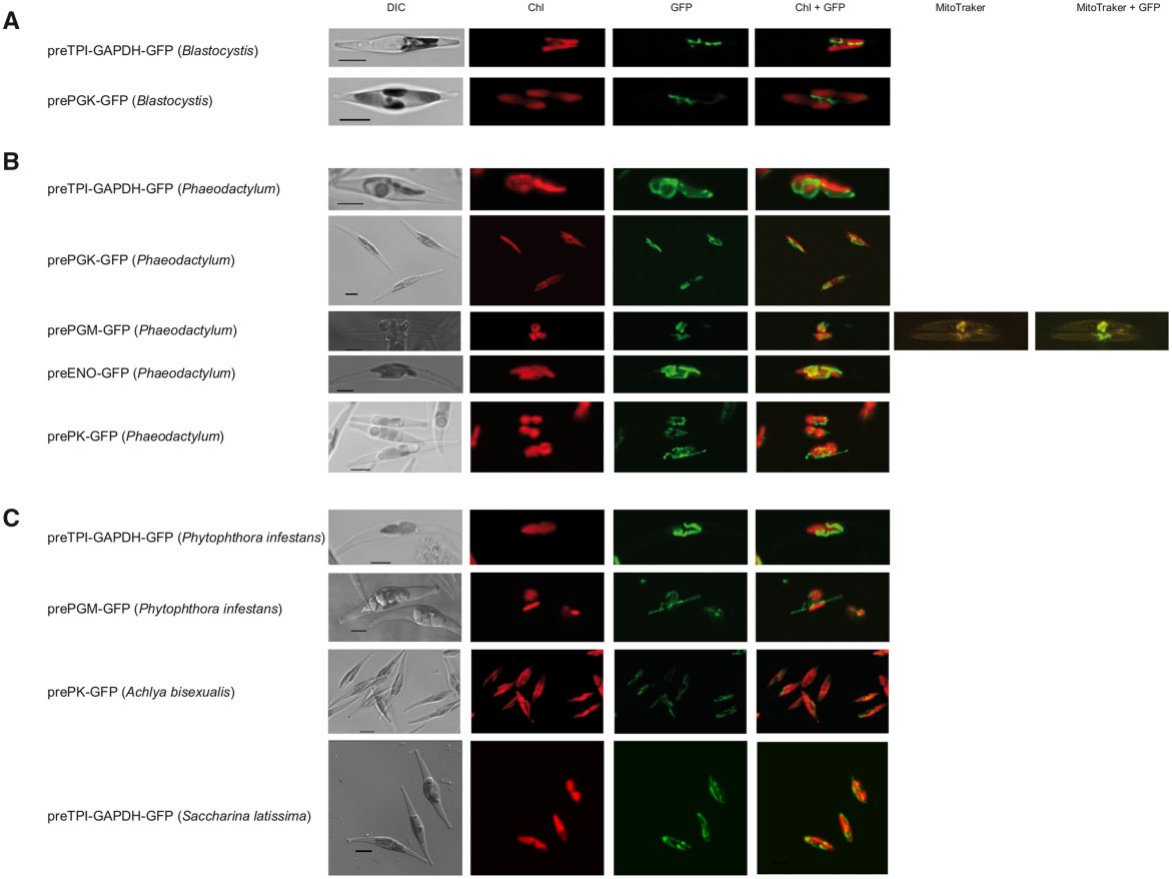
—Stramenopile glycolytic enzyme amino-terminal mitochondrial-like targeting signals are sufficient to target GFP to mitochondria in the diatom *Phaeodactylum tricornutum*. (*A*) The *Blastocystis* glycolytic enzymes TPI-GAPDH and PGK contain amino-terminal targeting signals that can target GFP to *P. tricornutum* mitochondria. (*B*) Amino-terminal extensions on TPI-GAPDH, PGK, phosphoglycerate mutase (PGM), enolase and pyruvate kinase (PK) from the diatom *P. tricornutum* were cloned in front of GFP and constructs used to transform *P. tricornutum*. (*C*) Amino terminal extensions on TPI-GAPDH and PGM from *Phytophthora infestans*, PK from *Achlya bisexualis* and TPI-GAPDH from *Saccharina latissima* were used as above to test for functionality of targeting information in *P. tricornutum.* DIC, Differential interference contrast microscopy. Chl, Chlorophyll *a* autofluorescence. GFP, Green fluorescent protein. Chl+GFP, Merged imaged showing the discrete (mitochondrial) localization of GFP. MitoTraker, MitoTraker Orange stain. MitoTraker+GFP, Merged image show considerable overlap of MitoTraker stain and GFP fluorescence. For the corresponding amino acid sequences used for GFP targeting, see supplementary file 1, Supplementary Material online. Scale bar 5 µm.

In order to test whether these predicated targeting signals are genuinely capable of guiding a protein to the mitochondria, we decided to try to target a reporter protein to these organelles. The predicted targeting signals were cloned in-frame upstream of the amino-terminus of the green fluorescent protein (GFP). The targeting constructs were used to transform *Phaeodactylum tricornutum*, a heterologous stramenopile alga and model system for stramenopile targeting (Gruber et al. 2007). The GFP reporter protein was targeted to discrete locations in the *Phaeodactylum* cells, typical of mitochondria in this diatom (see Ewe et al. 2018 for typical organellar localizations in *Phaeodactylum*; also see literature cited by Gruber et al. 2015) (fig. 2*A*). This suggests that these putative targeting signals are functional and sufficient to target a reporter protein to mitochondria of a heterologous host.

**Fig. 3.**
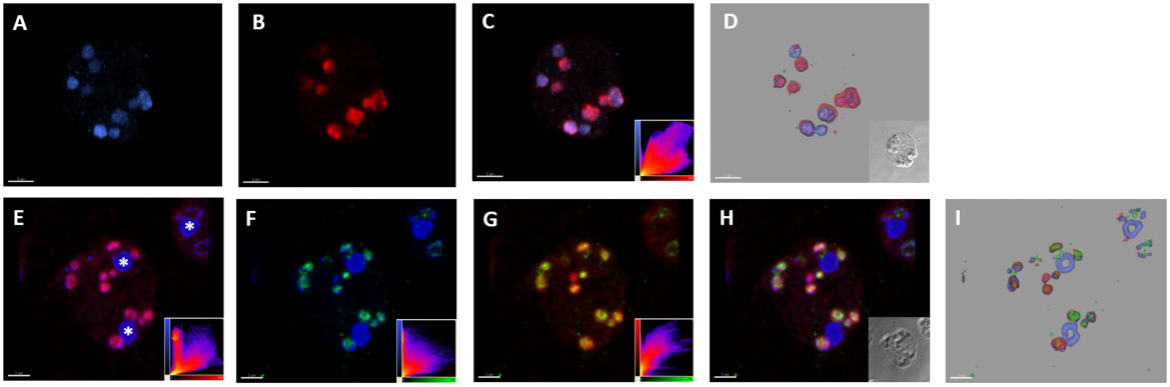
—The glycolytic enzymes TPI-GAPDH and PGK localize to mitochondria in the human parasite *Blastocystis.* Three-dimensional immunoconfocal microscopy reconstruction of optical sections (volume rendering) showing representative subcellular localization of PGK (blue) and TPI (red) in trophozoites (*A*–*D*, scale bar 3 µm). PGK (*A*) and TPI (*B*) volume signals show distinct distributions, consistent with localization within mitochondria, with considerable overlap. The merged image (*C*) provides a qualitative and the scatterplot (inset) of a quantitative measure of signal overlap. Colocalization of MitoTracker (red) and PGK (green) and DAPI (blue) in trophozoites (*E*–*I*). Merged images MitoTracker/DAPI (*E*) PGK/DAPI (*F*) and MitoTracker/PGK (*G*) and all three markers together (*H*) show considerable overlap with the exception of the DAPI signals for the nuclei (asterisks). Scatterplots (inset) give a quantitative measure of signal overlap for each merged pair of markers (*E*–*G*, scale bar 2 µm).

As heterologous targeting is not evidence that these proteins are actually localized in the mitochondrial organelles of its homologous host *Blastocystis*, we raised antibodies against both TPI-GAPDH and PGK. When these homologous antibodies were used together, it was clear that both TPI-GAPDH and PGK localise to the same structures in *Blastocystis* and the scatterplot analysis provides a clear quantitative measure of signal overlap (fig. 3*A*–*D*). To demonstrate these structures were the mitochondria in this organism, we used the PGK antibody together with the mitochondria-specific dye MitoTracker and the DNA dye DAPI. Both MitoTracker and DAPI have been used to label *Blastocystis* mitochondria previously (Stechmann et al. 2008). It is clear that PGK localises to the same structures as MitoTracker and DAPI (fig. 3*E*–*I*). The compartmentalized distribution of both TPI-GAPDH and PGK was clearly demonstrated in *Blastocystis* using confocal microscopy and three-dimensional rendering of optical sections confirming the mitochondrial localization of these glycolytic enzymes in this organism.

**Fig. 4.**
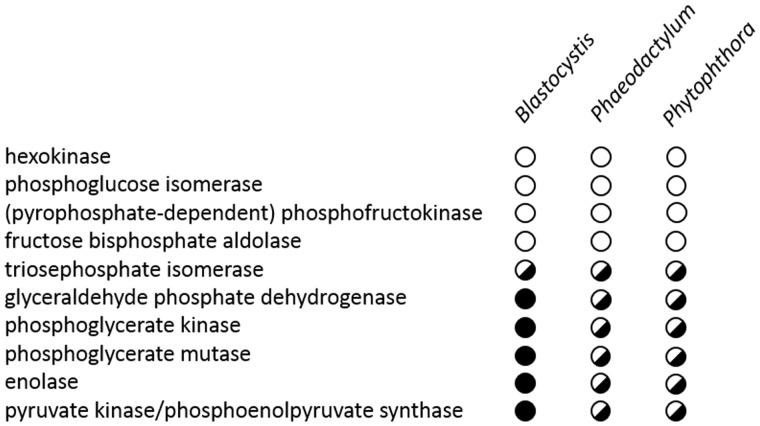
—Stramenopile glycolytic enzymes contain mitochondrial-like amino-terminal targeting sequences. Representative stramenopiles with whole genome data known are shown. Presence of mitochondrial-like targeting signal is shown with a filled circle, whereas open circle indicates no mitochondrial-like targeting signal. Where multiple isoforms with and without targeting signal exist, a half-filled circle is shown.

The unexpected mitochondrial localization of these three glycolytic enzymes in *Blastocystis* prompted us to check all glycolytic enzymes in this intestinal parasite for possible mitochondrial targeting signals. Interestingly, targeting signals were only observed on the enzymes that are involved in the pay-off phase of glycolysis but not in the investment phase (fig. 4).

Although three-dimensional reconstruction of our confocal microscopy data strongly indicated that these enzymes are indeed localized inside *Blastocystis* mitochondria (fig. 3), we additionally decided to thoroughly confirm these findings using classical enzyme assays following cellular fractionation. *Blastocystis* cells were broken and subsequently separated using differential centrifugations into nuclear, mitochondrial, lysosomal, and cytosolic fractions. Fractions were subsequently used in biochemical enzyme assays. These assays clearly showed that the activities of the five C3 enzymes are found in the mitochondrial pellet, whereas the five enzymes upstream in glycolysis are all confined to the soluble fraction (table 1). As it might be possible that the glycolytic enzymes were only attached to the surface of the mitochondria, as for instance in the case of hexokinase which is attached to the voltage-dependent anion channel (VDAC) in tumours (Lunt and Vander Heiden 2011), we tested the latency of enzymatic activities in the presence or absence of Triton X-100. The increase of measurable activity of the C3 but not the C6 glycolytic enzymes in the presence of detergent (table 2) strongly indicates the C3 enzymes are indeed retained within a membranous compartment.

**Table 1:** Pay-Off Phase Glycolytic Enzymes in *Blastocystis* are Found in the Mitochondrial Pellet

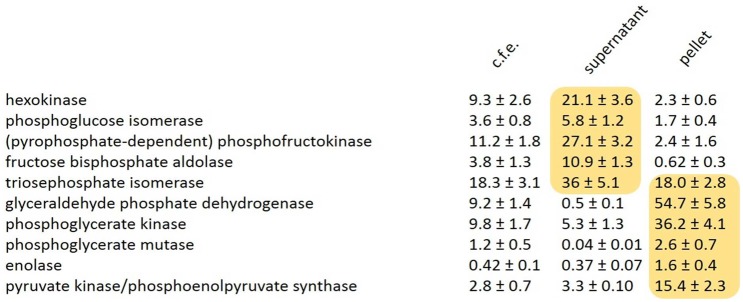

Note.—Activities of glycolytic enzymes from whole cell free extracts (c.f.e.) of *Blastocystis* suspended in phosphate buffered isotonic sucrose solution (pH 7.2). Cells were mixed at a ratio of two volumes of cells: three volumes of 0.5 mm glass beads and broken by three shakes of 1 min each at maximum speed on a bead beater (VWR mini bead mill homogenizer [Atlanta, GA, USA]). Cell-free extracts were subjected to increasing centrifugal force producing nuclear, mitochondrial (pellet), lysosomal and cytosolic (supernatant) fractions at 1,912 RCF_av_ for 5 min, 6,723 RCF_av_ for 15 min, 26,892 RCF_av_ for 30 min, respectively. Enzyme activities are the average of three determinations ± SD. *1 enzyme unit (EU) is the amount of enzyme that converts 1 µmole substrate to product per minute. The yellow box indicates the site of major activity (or in the case of triosephosphate isomerase, the dual localization).

When proteolytic enzymes were used it was clear that these only affected the measured activity in the presence of the detergent Triton X-100 (table 3). This clearly demonstrates that the five C3 glycolytic enzymes in *Blastocystis* are protected by a membrane and reside inside the mitochondria and not on the outside of the organelle, as observed in certain tumours (Lunt and Vander Heiden 2011) or some proteomics studies (Giege et al. 2003; but see Smith et al. 2007). This is the first genuine confirmation of true glycolytic enzyme activity inside mitochondria.

**Table 2:** Latency of ML Organelles with Respect to Glycolytic Enzymes

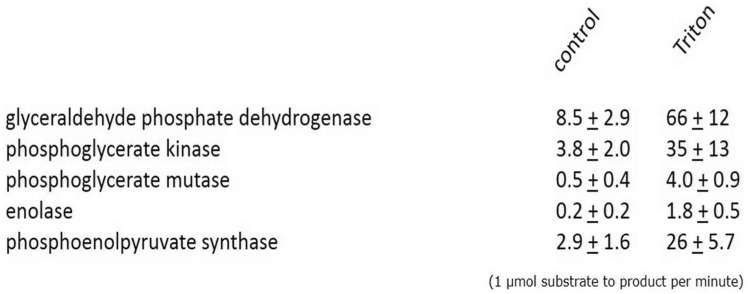

Note.—Glycolytic enzymes localizing to the mitochondrial-like organelle were assayed in 225 mM sucrose buffered solutions complete with substrate(s) and monitored on a spectrophotometer for 15 min. Triton X-100 (0.1%) was added and the wavelength monitored for a further 15 min. Results are presented as the mean ± SD of triplicate experiments.

**Table 3:** *Blastocystis* Glycolytic Enzymes are Protected by a Membrane

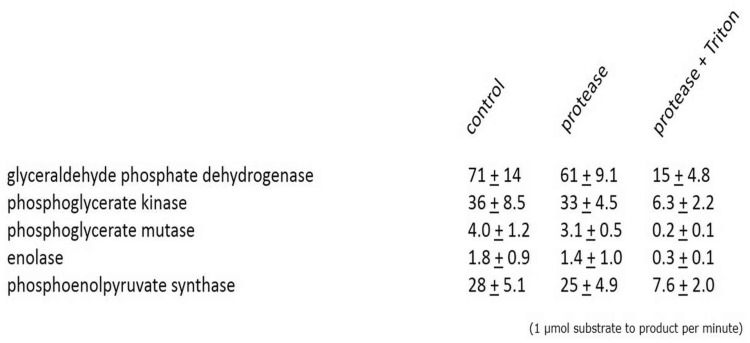

Note.—Control: Mitochondrial fractions incubated without proteolytic enzymes. Protease: Mitochondria incubated in 225 mM sucrose buffer at 25 °C containing 500 U bovine pancreas trypsin, 10 U papaya latex papain and 250 U porcine pepsin for 15 min. Protease + Triton: Mitochondrial fractions containing proteolytic enzymes and 1% Triton X-100 incubated for 15 min at 25 °C. Samples were centrifuged (14,000 × g) for 2 min and resuspended in fresh sucrose buffer without proteolytic enzymes prior to assay.

As some of us previously reported the mitochondrial localization of the TPI-GAPDH fusion protein in a related stramenopile (Liaud et al. 2000), we wondered whether mitochondrial targeting of glycolytic enzymes is more widespread in this group of organisms. When querying other stramenopile genomes, we noticed the widespread presence of mitochondrial targeting signals on glycolytic enzymes within the whole group. Here, as with *Blastocystis*, only enzymes of the C3 part of glycolysis contain mitochondrial targeting signals (figs. 1*B* and 4).

**Fig. 5.**
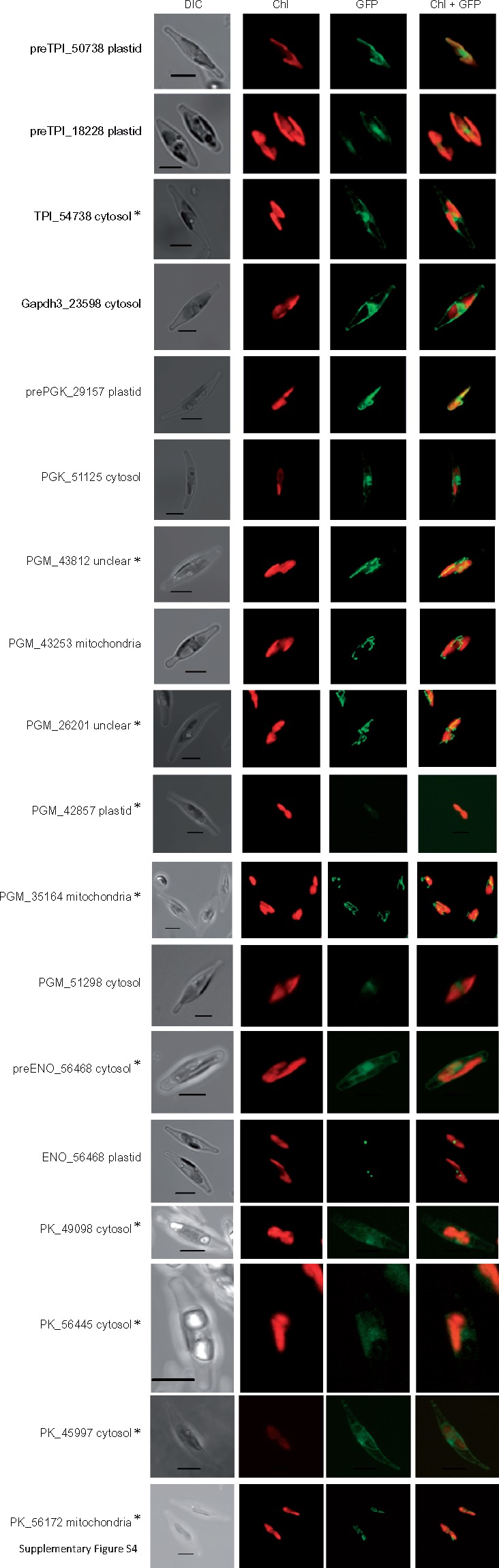
—*Phaeodactylum tricornutum* contains, similar to some other stramenopiles, multiple isoforms for the C3 part of glycolysis. The localization for all isoforms was tested via GFP-fusion constructs. A “pre” suffix means that the predicted targeting signal was used; if the suffix is missing the full length of the respective sequence was fused to GFP. The number is the JGI Protein ID and the result of each localization is mentioned. A star (*) marks images were a maximum intensity projection from a Z-Stack was used. Unclear indicates localization not possible to identify. TPI = Triosephosphate isomerase, GAPDH = glyceraldehyde-3-phosphate dehydrogenase, PGK = phosphoglycerate kinase, PGM = phosphoglycerate mutase, ENO = enolase, PK = pyruvate kinase. DIC, Differential interference contrast microscopy. Chl, Chlorophyll *a* autofluorescence. GFP, Green fluorescent protein. Chl+GFP, Merged imaged showing the discrete localization of GFP compared with Chlorophyll autoflourescence. For the corresponding amino acid sequences used for GFP targeting, see supplementary file 2, Supplementary Material online. Scale bar 5 µm.

**Fig. 6.**
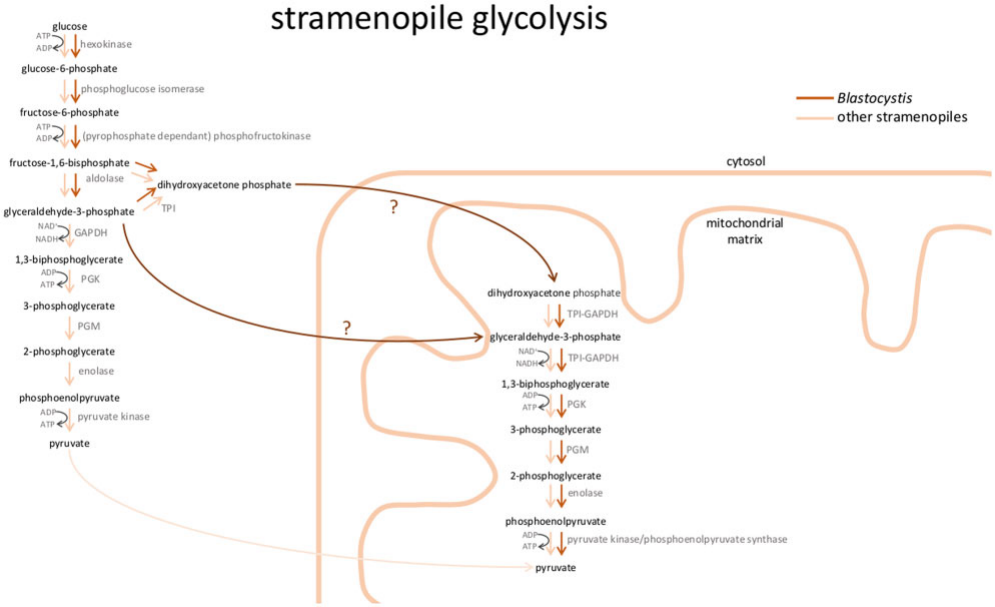
—Stramenopile glycolysis. Localization of glycolytic enzymes in the stramenopiles is distributed between cytosol and mitochondria based on our cell biological and biochemical data. For the intestinal parasite *Blastocystis*, the pay-off phase is solely localized to the mitochondrial matrix, whereas for all other studied stramenopiles the pay-off phase is found in the cytosol as well as the mitochondrion. It is not known which intermediary glycolytic substrate is transported into mitochondria (indicated by question marks). Flow is shown in the direction of pyruvate only. TPI, triosephosphate isomerase; GAPDH, glyceraldehyde phosphate dehydrogenase; PGK, phosphoglycerate kinase; PGM, phosphoglycerate mutase.

To test for functionality, we also tested all predicted mitochondrial targeting signals from *Phaeodactylum* C3 glycolytic enzymes. The targeting signals were fused to GFP and their cellular location was determined (fig. 2*B*). As with *Blastocystis*, all constructs were targeted to the mitochondria suggesting these are genuine mitochondrial targeting signals in vivo. In addition, we also tested mitochondrial targeting signals found on glycolytic enzymes of the oomycete pathogen *Phytophthora infestans*, the water mould *Achlya bisexualis* and the multicellular brown alga *Saccharina latissima*, commonly known as kelp (fig. 2*C*). In all cases, these targeting signals targeted GFP into the mitochondria of the diatom suggesting that mitochondrial localization of the C3 part of glycolysis is a more general feature in the stramenopiles. However, for some organisms we also detected nontargeting signal bearing glycolytic enzymes suggesting that these cells possibly have a branched glycolytic pathway (see figs. 5 and 6).

The mitochondrial proteome has a complex and contested evolutionary past (van der Giezen 2011; Ku et al. 2015; Pittis and Gabaldon 2016; Martin et al. 2017), and we wondered if glycolytic enzymes targeted to mitochondria might have different evolutionary origins than those that operate in the cytosol. Sequences of all glycolytic enzymes from *Phaeodactylum tricornutum* and *Blastocystis* ST1, strain NandII, were used as seeds in BlastP searches in the nonredundant database at the NCBI (Altschul et al. 1990). We were especially interested to identify all sequences in the SAR supergroup (Adl et al. 2012) (Stramenopiles, Alveolates, and Rhizaria). In addition, representatives from other eukaryotic groups and, if required, closely related bacterial sequences were added. Phylogenetic analysis of all glycolytic enzymes does not seem to be able to support either hypothesis of different evolutionary origins of cytosolic versus mitochondrial glycolytic enzymes (supplementary fig. S1*A*–*F*, Supplementary Material online).

## Discussion

Eukaryotes evolved from a symbiosis between an archaeal host and a bacterial endosymbiont that became the mitochondrion (van der Giezen 2011; Martin et al. 2015). Although many different hypotheses have been posited over the years, they principally boil down to two scenarios. The phagotropic origin of eukaryotes suggests they evolved gradually from a less complex prokaryote and once phagotrophy had evolved, the mitochondrial endosymbiosis was possible (see O'Malley 2010). The syntrophic eukaryotic origin suggests the establishment of the mitochondrial endosymbiont was the same event as the origin of eukaryotes (see Martin et al. 2015). Arguments have been put forward for and against either scenario and it seems that biochemical/physiological arguments favor a synthrophic origin and cell biological/morphological arguments favor a phagotrophic origin. Both scenarios seem to agree that the host was archaeal and the endosymbiont bacterial (Martin et al. 2015; Roger et al. 2017). The subsequent replacement of the host’s gene repertoire encoding metabolic capacity has been explained by endosymbiotic gene transfer (reviewed in Timmis et al. 2004) and the resulting chimeric nature of eukaryotes had been noticed earlier (Rivera et al. 1998). The nature of the mitochondrial endosymbiont has long been understood to be alpha-proteobacterial (Gupta 1995) but only recently have studies zoomed in on the more precise affiliations of the archaeal host (Cox et al. 2008; Williams et al. 2013; Martin et al. 2015; Eme et al. 2017; Zaremba-Niedzwiedzka et al. 2017). A recent study suggests that mitochondria are perhaps ancestral to alpha-proteobacteria, but does not exclude an alpha-proteobacterial origin (Martijn et al. 2018). A few billion years of independent evolution of the endosymbiont’s lineage and widespread bacterial lateral gene transfer (even predating the mitochondrial symbiosis) can explain that not all eukaryotic metabolic proteins have a clear alpha-proteobacterial evolutionary signal. Despite all this, glycolytic enzymes of eukaryotes do all cluster with bacterial homologues in phylogenetic trees (supplementary fig. S1, Supplementary Material online; Martin et al. 1993; Martin and Herrmann 1998; Esser et al. 2004). It is indeed implicit of eukaryotic origin theories (Martin and Müller 1998; Martin et al. 2015) that glycolysis was originally acquired from the mitochondrial endosymbiont. It is therefore interesting to consider whether the mitochondrial targeting of glycolytic enzymes in stramenopiles represents an ancestral or a derived state for eukaryotes. The deep branches of the eukaryotic tree are not known with certainty, but there is substantial phylogenomic support for the grouping of stramenopiles with alveolates and rhizarians to form the “SAR” supergroup (Burki et al. 2008). Intriguingly, predicted mitochondrial targeting has been reported for several glycolytic enzymes—including TPI-GAPDH fusion proteins—in members of the cercozoa, a group of rhizarians (Nakayama et al. 2012); the distantly related apusozoan *Thecamonas trahens* also encodes a TPI-GAPDH fusion protein (Nakayama et al. 2012). Taken together, these data raise the possibility that at least some of the latter steps of glycolysis may have occurred in the mitochondria of the SAR common ancestor (Nakayama et al. 2012). However, these inferences are currently based on a very limited sample of SAR diversity, and testing hypotheses about the localization of glycolysis in early eukaryotes will require both more genomes and more of the experimental characterization that we report here.

Evolution of mitochondrial protein targeting was a requirement for the successful integration of the mitochondrial endosymbiont and should have happened at least concomitant with endosymbiotic gene transfer if those gene products had to function in the newly formed organelle (comparison to more recently evolved host/symbiont systems suggests that the first proteins that are targeted to an endosymbiont in fact do not originate from the endosymbiont and that the evolution of protein targeting precedes the direct transfer of endosymbiont genes to the host nucleus [Nowack 2014]). Mitochondrial targeting signals do not conform to a strict consensus sequence and secondary structure is a key factor in their functionality (Schatz and Dobberstein 1996). These presequences form amphipathic alpha helices with alternating hydrophobic and positively charged amino acids (Allison and Schatz 1986; von Heijne 1986; Roise et al. 1988). Mitochondrial targeting sequences can arise randomly (Baker and Schatz 1987), exist in bacteria (Lucattini et al. 2004) and can be acquired by DNA recombination or exon shuffling (Wischmann and Schuster 1995; Long et al. 1996; Kubo et al. 1999). The predicted presequences for the stramenopiles are in the size range of known mitochondrial targeting signals (von Heijne et al. 1989) and are also enriched in alanine, leucine, serine and arginine (Neupert and Herrmann 2007). Organellar targeting signals for mitochondrial remnants such as the mitochondrial organelle in *Blastocystis* tend to be shorter than but not as short as those found for *Trichomonas* hydrogenosomes (van der Giezen et al. 2005; Garg et al. 2015). However, there do seem to be some characteristic features even for these hydrogenosomal presequences with often a leucine at the second position and an arginine two places before the cleavage site (Bradley et al. 1997; van der Giezen et al. 1998).

It is difficult to conclusively determine the selective advantage, if any, for the retargeting or the conservation of glycolysis to/in stramenopile mitochondria. In *Blastocystis*, similar to many parasitic eukaryotes (Mertens 1993), two key glycolytic enzymes have been replaced by pyrophosphate using versions. Normally, the reactions catalysed by phosphofructokinase and pyruvate kinase are virtually irreversible. However, the reactions performed by diphosphate-fructose-6-phosphate 1-phosphotransferase and phosphoenolpyruvate synthase (pyruvate, water dikinase) are reversible, due to the smaller free-energy change in the reaction. As *Blastocystis* is an anaerobe and does not contain normal mitochondrial oxidative phosphorylation (Stechmann et al. 2008; Gentekaki et al. 2017), any ATP not invested during glycolysis might be a selective advantage. However, in the absence of these irreversible control points there is a risk of uncontrolled glycolytic oscillations (Chandra et al. 2011). Separating the investment phase from the pay-off phase by the mitochondrial membranes might therefore prevent futile cycling. However, as not all stramenopiles use pyrophosphate enzymes, this cannot be the whole explanation.

Similarly to the peculiarity of pyrophosphate utilization in *Blastocystis*, diatoms also show metabolic peculiarities that are not shared with other organisms (Gruber and Kroth 2017). One such peculiarity is the presence of an Entner–Doudoroff pathway in the mitochondria of *P. tricornutum* (Fabris et al. 2012). This pathway, like glycolysis, degrades glucose to pyruvate. However, the net ATP yield of the Entner–Doudoroff pathway is lower (one ATP per glucose) and the two reducing equivalents that are formed are one NADH and one NADPH per glucose. The degradation of glyceraldehyde 3-phosphate in the Entner–Doudoroff pathway uses identical reaction steps as the glycolysis. Mitochondrial glycolysis therefore might be a complement of the mitochondrial Entner–Doudoroff pathway in *P. tricornutum* (and other photosynthetic stramenopiles with an Entner–Doudoroff pathway) (Fabris et al. 2012). However, we did not find evidence for an Entner–Doudoroff pathway in nonphotosynthetic stramenopiles, so again, this explanation might not be valid for all stramenopiles with mitochondrial glycolysis.

Glycolysis depends on recycling of the reducing equivalents that are formed in the GAPDH reaction (in which NAD^+^ is reduced to NADH). How NAD+ is regenerated depends on the presence of oxygen. Under anoxic conditions, pyruvate usually is reduced in a fermentation which recovers oxidized NAD^+^ (most commonly lactic acid or ethanol fermentation). Under aerobic conditions, the reducing equivalents are transferred to O_2_ in the mitochondrial respiratory electron transport chain. In organisms that operate glycolysis exclusively in the cytosol, NAD^+^/NADH apparently cannot be transported directly into mitochondria. Instead two shuttle systems, the glycerol phosphate shuttle and the malate-aspartate shuttle, lead to indirect exchange of reducing equivalents between cytosol and mitochondria. To release reducing equivalents directly in the mitochondrial matrix where they can be accepted by the respiratory electron transport chain without the need of a shuttle system seems an elegant solution. Similarly, if the redox shuttle system between cytosol and mitochondrial matrix is absent, it also makes sense that the NADPH generating glucose-6-phosphate-dehydrogenase reaction in the above mentioned Entner–Doudoroff pathway in photosynthetic stramenopiles takes place in the mitochondria.

The malate-aspartate shuttle requires a cytosolic malate dehydrogenase (MDH). *P. tricornutum* does not possess a cytosolic MDH (Ewe et al. 2018), which might also suggest an absence of a malate-aspartate shuttle in this diatom. However, if difficulties in redox shuttling would require the redox reactive steps to occur in the mitochondria, this would not explain mitochondrial glycolysis in *Blastocystis*, an organism that does not rely on oxidative ATP generation. Furthermore, physiological data suggests that in diatoms, considerable shuttling of reducing equivalents from the plastid to the mitochondria may occur as a measure to prevent the formation of reactive oxygen species at the photosystems when excessive excitation energy is absorbed (Allen et al. 2008; Bailleul et al. 2015). These findings, and also the presence of unusual transport proteins for nucleotides (Ast et al. 2009; Chu et al. 2017), do not support the hypothesis of a lack of efficient shuttling, but underline the importance of stramenopile mitochondria as electron sinks in the recycling of electron acceptors that are reduced either in the mitochondria (in the mitochondrial pay-off phase of glycolysis or in the above-mentioned Entner–Doudoroff pathway) or in other compartments (in the cytosolic part of glycolysis or in the photosynthetic electron transport chain in the plastids).

Recently, Abrahamian et al. (2017) reported similar findings to ours and used GFP-tagged proteins in *P. infestans* to demonstrate the mitochondrial localization of glycolytic enzymes. They also report the targeting of several steps of a serine anabolic pathway to *P. infestans* mitochondria and suggested the shared 3-phosphoglycerate intermediate would be the *raison d'être* for the mitochondrial glycolysis (Abrahamian et al. 2017).

All the points discussed above might indeed provide several possible physiological explanations for the observed mitochondrial glycolysis in stramenopiles, but unfortunately do not answer the question whether mitochondrial glycolysis is a primary or secondary state in these groups of eukaryotes.

The end-product of glycolysis, pyruvate, is transported into mitochondria via a specific mitochondrial transporter that has only recently been identified (Herzig et al. 2012) and that is absent from the *Blastocystis* genome (Gentekaki et al. 2017). The translocation of the C3 part of glycolysis into mitochondria would necessitate a novel transporter (presumably for triose phosphates). The identification and characterization of such a transporter would open up new possible drug targets against important pathogens. Examples include *Phytophthora infestans*, the causative agent of late potato blight, which has a devastating effect on food security, but also fish parasites such as *Saprolegnia parasitica* and *Aphanomyces invadans.* Both have serious consequences for aquaculture and the latter causes epizootic ulcerative syndrome, an OIE listed disease (Jiang and Tyler 2012; Stentiford et al. 2014). Our recent genome analysis of *Blastocystis* identified several putative candidate transporters lacking clear homology to nonstramenopile organisms (Gentekaki et al. 2017). Such a unique transporter would not be present in the host (including humans) and could be exploited to prevent, or control, disease outbreaks that currently affect food production while the world population continuous to increase (FAO 2009).

## Conclusion

Taken together, our results show that glycolysis, contrary to the textbook view on well-investigated model organisms, not only occurs in the cytosol, but also occurs in the mitochondria. All tested stramenopiles show evidence of the second half of glycolysis taking place in the mitochondria and the cytosol, with the exception of the human pathogen *Blastocystis*, in which the second half of glycolysis occurs exclusively in the mitochondria. Mitochondrial glycolysis therefor seems to be a common feature of the stramenopiles, despite the considerable metabolic and physiological diversity within this group. Although it remains unclear whether this feature is ancestral or derived, our findings show that the intracellular distribution of even the most basic metabolic pathways is variable between the different groups of eukaryotes.

## Supplementary Material

Supplementary Data
