# Supplementary material for: Mitochondrial Glycolysis in a Major Lineage of Eukaryotes
Source: Genome Biol Evol. 2018 Jul 30;10(9):2310–25. doi: 10.1093/gbe/evy164 (PMC6198282; doi:10.1093/gbe/evy164)
Supplement: Supplementary Data [file evy164_supp.zip › evy164_Supp/Supplementary Figure 180503.pdf]

Supplementary figure legends

Figure S1. Phylogenetic analysis of glycolytic enzymes of the pay-off phase. A. Triosephosphate isomerase (TPI), 77 sequences and 167 amino acid positions were used to calculate the tree. Bacterial sequences were used as outgroup. B. Glyceraldehyde-3-phosphate dehydrogenase (GAPDH), only GAPDH from the C –type are illustrated in the tree. 96 sequences and 266 amino acid positions were used to calculate the tree. One branch caused long branch attraction (LBA) artifact and therefore is shortened in the figure. C. Phosphoglycerate kinase (PGK), 66 sequences and 360 amino acid positions were used to calculate the tree. D. Phosphoglycerate mutase (PGM), 96 sequences and 199 amino acid positions were used to calculate the tree. E. Enolase (ENO), 80 sequences and 345 amino acid positions were used to calculate the tree. F. Pyruvate kinase (PK), 80 sequences and 287 amino acid positions were used to calculate the tree. Values at nodes: posterior probabilities (P.P. >0.5) / rapid bootstrap values (BS>30%). Species name in bold = localization experimental proof (arrow = in this study, star Liaud *et. al* (2000)). TargetP analysis: M = mTP = mitochondria (bold indicates scores > 0.700), O = other, SP = signal peptide, C = cTP = chloroplast only for Viridiplantae, Rhodophyta and Glaucocystophyceae plant results was taken if non-plant results differ. N.D.: sequences not analyzed; not complete at N-terminus or start methionine is missing (proof by an alignment). Colour code: Viridiplantae, Stramenopiles, Alveolata, Rhizaria, Rhodophyta, Cryptophyta, Haptophyceae, Bacteria, other Eukaryota, Archaea, Cyanobacteria, Euglenozoa.

A. TPI

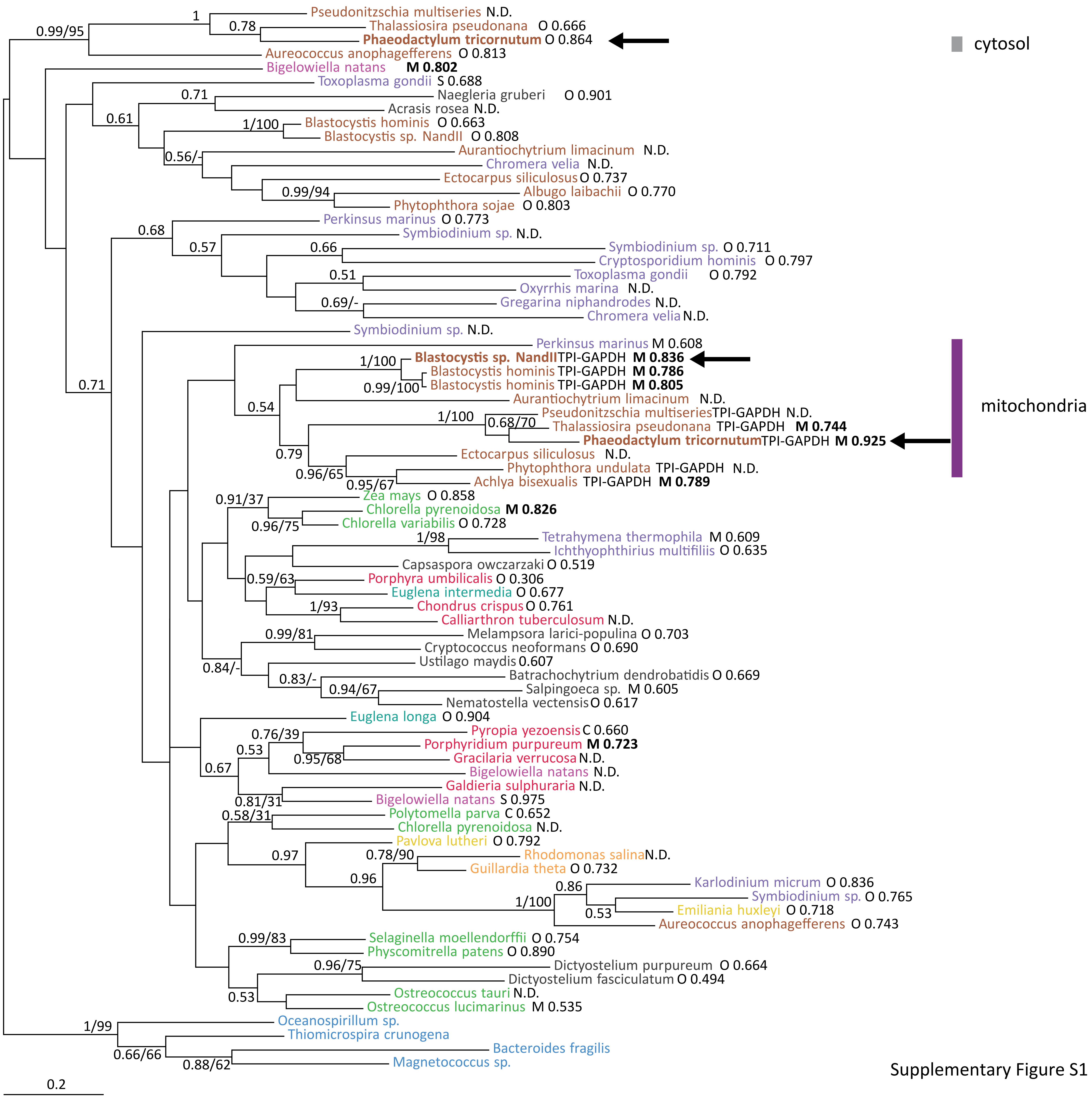

Supplementary Figure S1

B. GAPDH

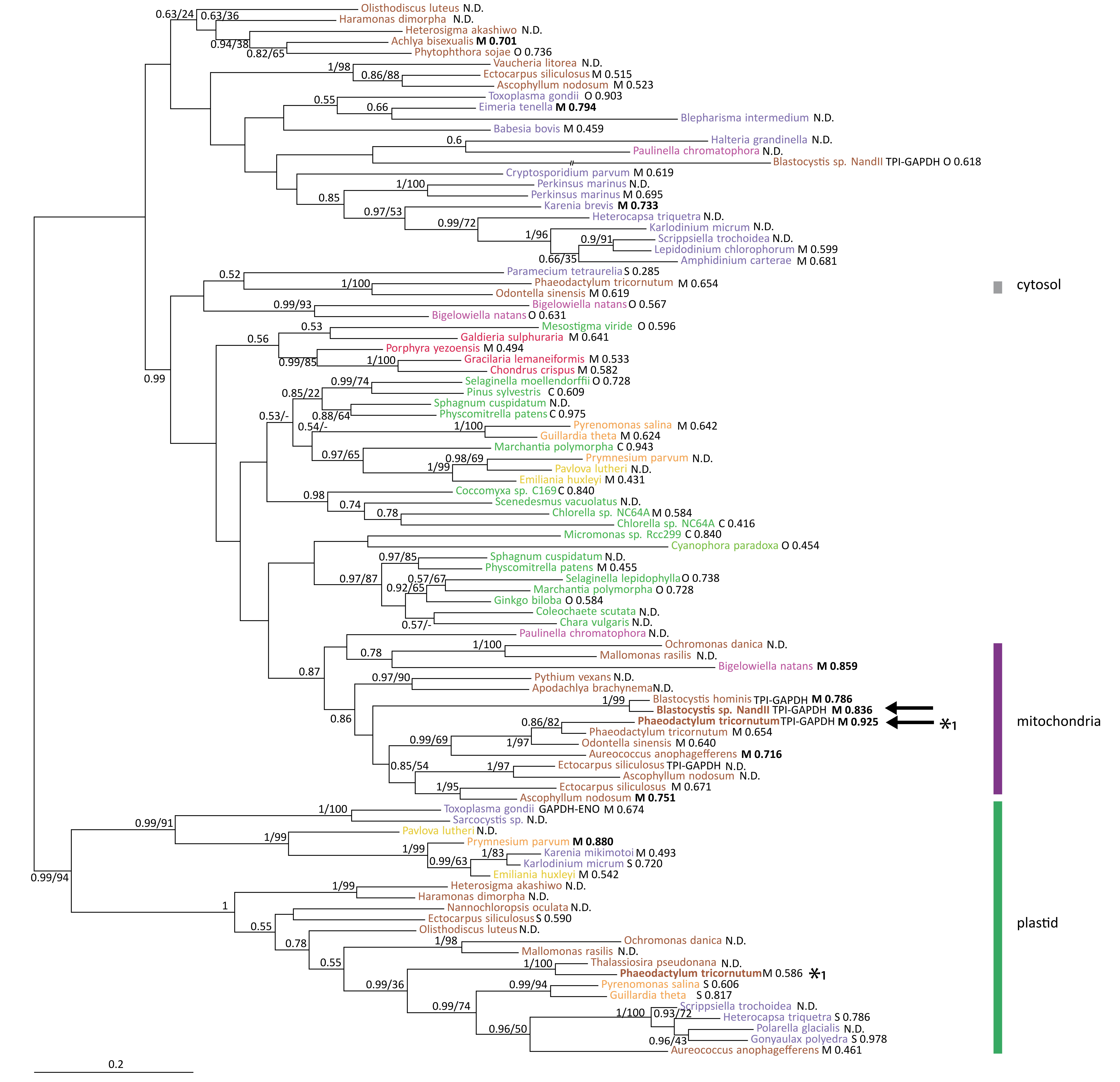

Supplementary Figure S1

C. PGK

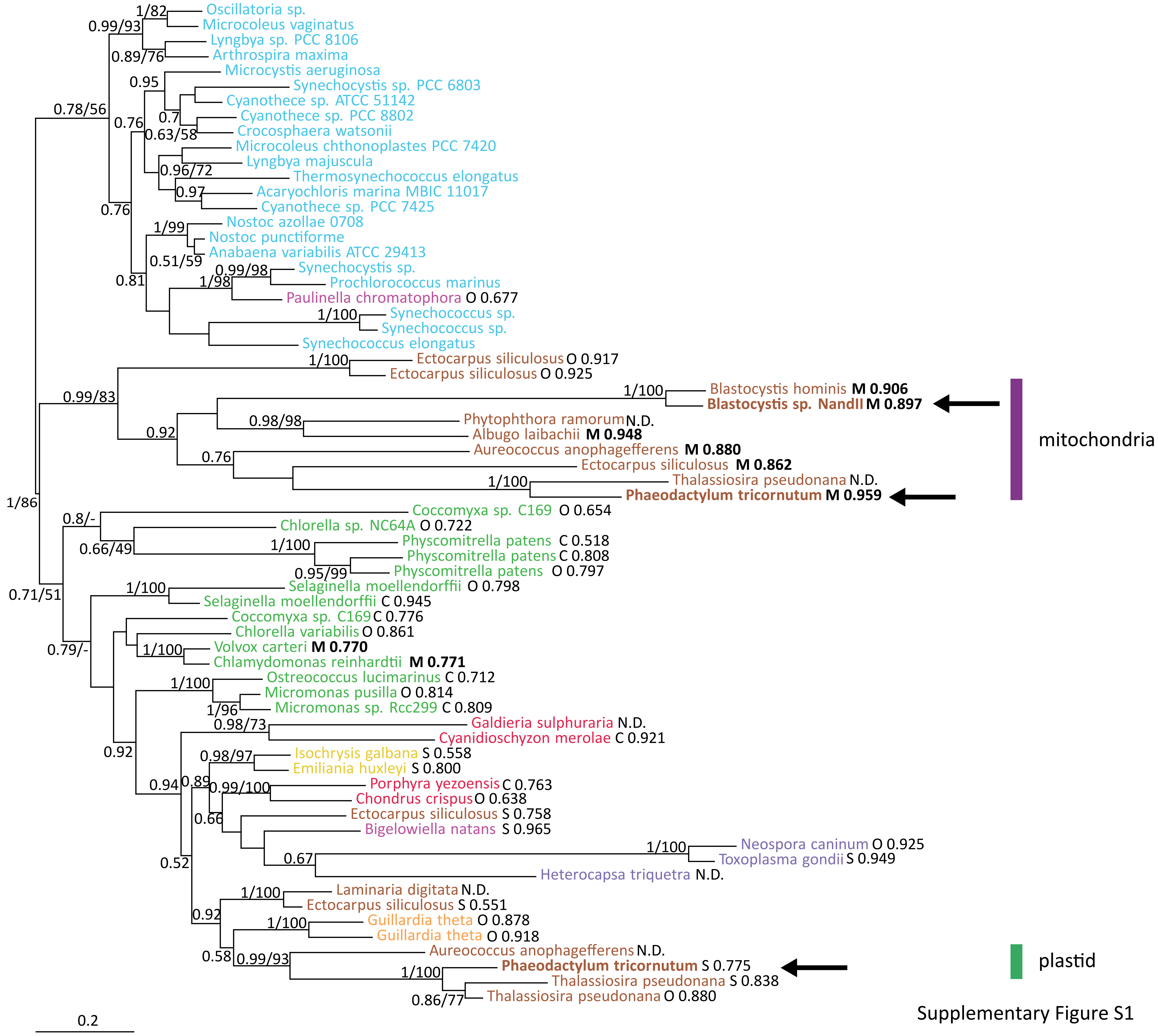

Supplementary Figure S1

D. PGM

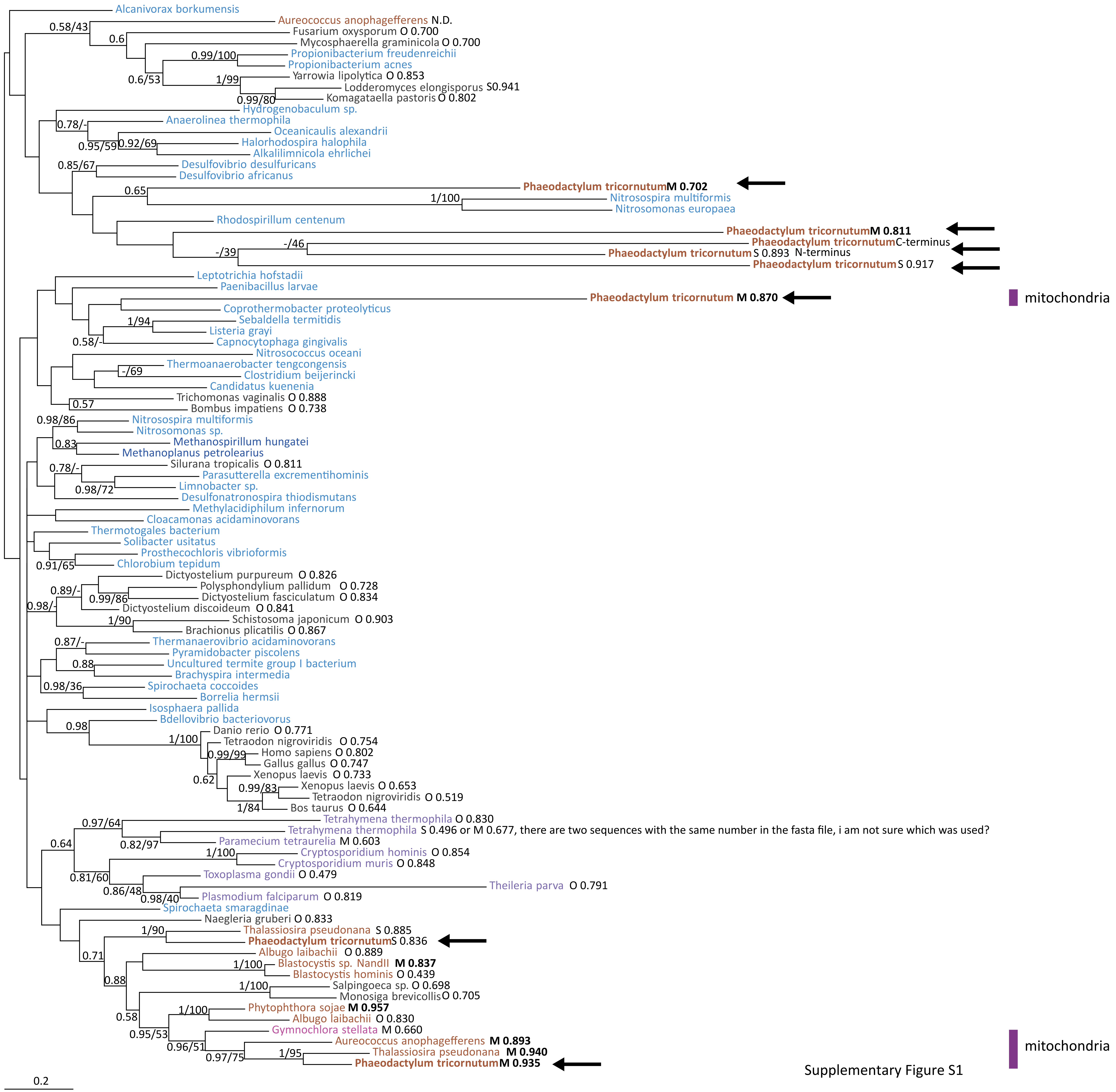

Supplementary Figure S1

E. ENO

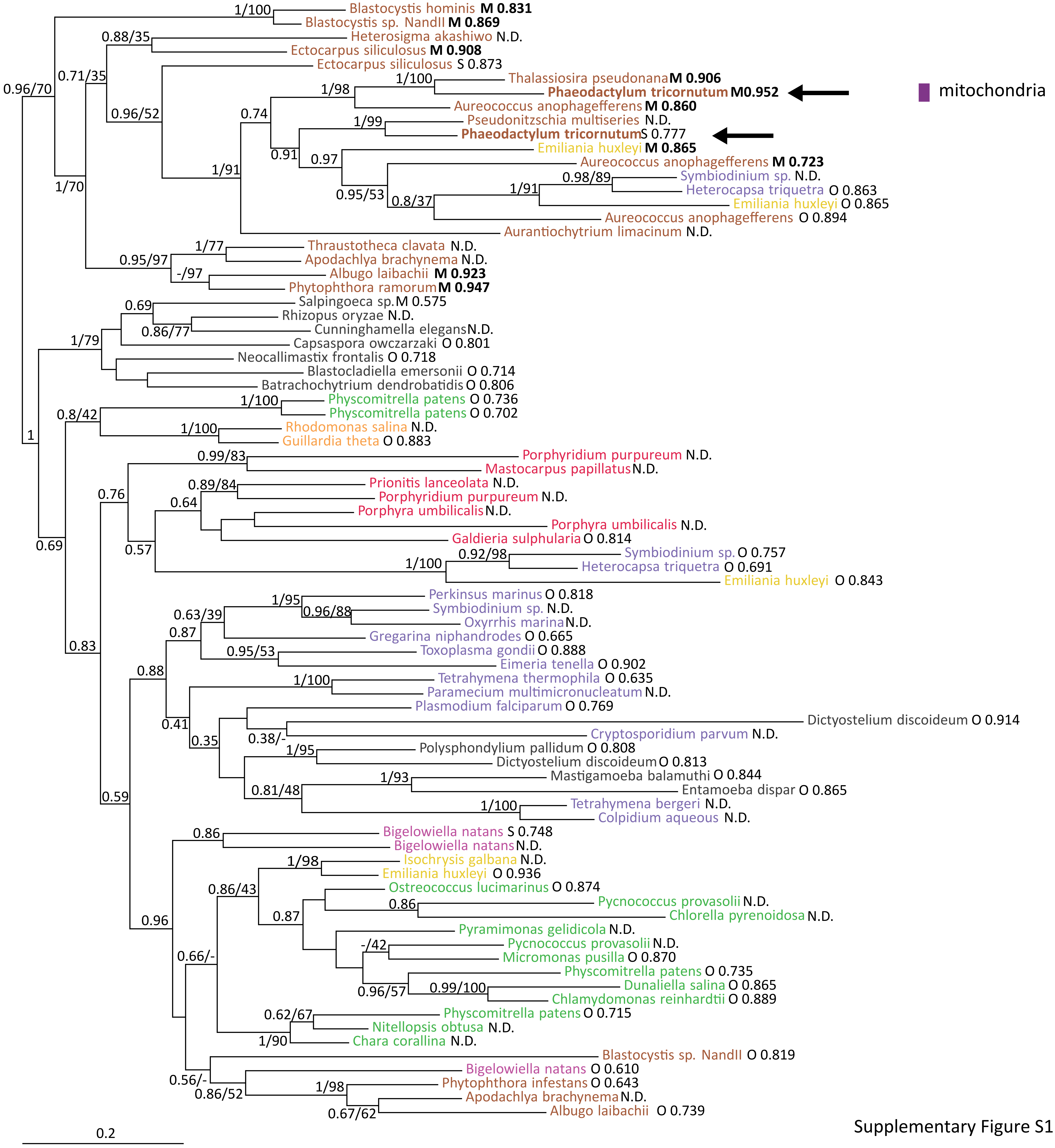

Supplementary Figure S1

F. PK

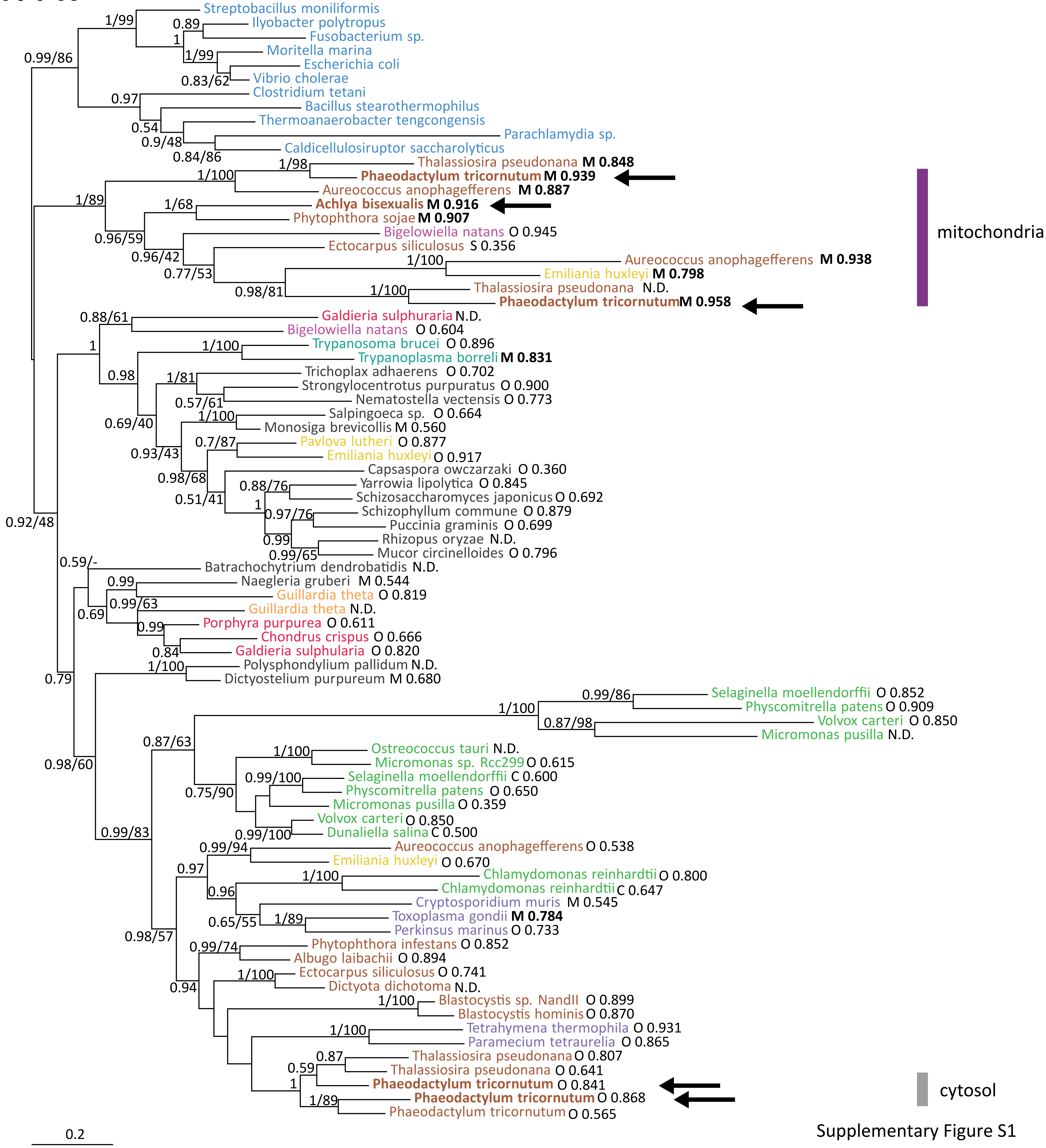

Supplementary Figure S1
