# Supplementary material for: Mitochondrial Glycolysis in a Major Lineage of Eukaryotes
Source: Genome Biol Evol. 2018 Jul 30;10(9):2310–25. doi: 10.1093/gbe/evy164 (PMC6198282; doi:10.1093/gbe/evy164)
Supplement: Supplementary Data [file evy164_supp.zip › evy164_Supp/Supplementary file 1 180503.pdf]

Amino acid sequences of mitochondrial targeting sequences used in GFP targeting experiments as seen in Figure 2.

A.

> preTPI-GAPDH-GFP (*Blastocystis*) (OAO12326)

MLSRSSVIARSFGSAARKL

>prePGK-GFP (*Blastocystis*) (OAO15536)

MLSAFSKRLFSTGRTVN

B.

> preTPI-GAPDH-GFP (*Phaeodactylum*) (NCBI AF063804)

MLASSRTAAASVQRMSSRAFHASSLTEARKFFVGGNWKCNGS

>prePGK-GFP (*Phaeodactylum*)(JGI 48983)

MLFRMLTSTALRRSPVTTSLTCCCKANAFAVRIRSFHAAPVIQAKMTVEQLAQQ

>prePGM-GFP (*Phaeodactylum*)(JGI 33839)

MFAVSRSSFLLATRVKTLRSFAAVQAADKHTLVLLRHGESTWNLENKFTGWYDCP

>preENO-GFP (*Phaeodactylum*)(JGI 1572)

MMWSRPVLRRNISTTRASSSSRRFLSAITGVHGREIDSRGNPTVEVDVTTAQGT

>prePK-GFP (*Phaeodactylum*) (JGI 49002)

MMRSFLRHAQGRACAQHLRTIGTLRLNQMPVTGA

C.

>preTPI-GAPDH-GFP (*Phytophthora infestans*) (NCBI X64537)

MSFRQVFKTQARHMSSSSRKFFVGGNWKCNGSLGQAQELVGMLNTA

>prePGM-GFP (*Phytophthora infestans*) (PfGD Pi_011_55705_Feb05.seq)

MVLALRRPLAISSRVANRSLGMLRQQQKAMKHTHTLVLIRHGESEWNKKNLFTGWYDVQLSEKGNKEA

>prePK-GFP (*Achlya bisexualis*) (NCBI AAU81895)

MLARSLRSRAVRSFARGLSNKPSKNDAFSMT

>preTPI-GAPDH-GFP (*Saccharina latissima*) (NCBI ABU96661)

MFSAALSAAGAKAPSAARGFASSASRMSGRKFFVGGNWKCNGS
