# Supplementary material for: Mitochondrial Glycolysis in a Major Lineage of Eukaryotes
Source: Genome Biol Evol. 2018 Jul 30;10(9):2310–25. doi: 10.1093/gbe/evy164 (PMC6198282; doi:10.1093/gbe/evy164)
Supplement: Supplementary Data [file evy164_supp.zip › evy164_Supp/Supplementary file 2 180503.pdf]

Phaeodactylum tricornutum **amino acid sequences used in GFP targeting experiments as seen in Figure 5.**

>preTPI_50738 plastid (pre-sequence) (JGI 50738) plastid

MTGDSTSLLDLISPDRERPQRKEPSRWIAFSVFPFVRFIPEAFATRLPYSIVMKFLALSVAALISSATAFAPTFR
GSPASTTASTTSLAARKPFISGNWKLN

>preTPI_18228 plastid (pre-sequence) (JGI 18228) plastid

MKFLALSVAALISSATAFAPTFRGSPASTTASTTSLAARKPFISGNWKLN

>TPI_54738 cytosol (242 Amino acids) (JGI 54738) cytosol

MPRPDGSSTPAAEGERKYLVAGNWKCNGTLASNEELVKTFNEAGPIPSNVEVAICCPSLYLPQLLSSLRDDIQIG
AQDCGVNDKNGAFTGEIGAFQIKDIGCDWVIIGHSERRDGFEMPGETPDLCAKKTRVAIDAGLKVMFCIGEKKEQ
REDGTTMDVCASQLEPLAAVLTESDWSSIAIAYEPVWAIGTGLTATPEMAQETHASIRDWISQNVSADVAGKVRI
QYGGSMKGANAKDLLEQ

>Gapdh3_23598 cytosol (full length) (JGI 23598) cytosol

MPVKCLVNGFGRIGRLCFRYAWDDPELEIVHVNDVCSCESAAYLVQYDSVHGTWSKSVVAAEDSQSFTVDGKLVT
FSQEKDFTKIDFASLGVDMVMECTGKFLTVKTLQPYFGMGVKQVVVSAPVKEDGALNVVLGCNHQKLTTDHTLVT
NASCTTNCLAPVVKVIQENFGIKHGCITTIHDVTGTQTLVDMPNTKKSDLRRARSGMTNLCPTSTGSATAIVEIY
PELKGKLNGLAVRVPLLNASLTDCVFEVNKEVTVEEVNAALKKASESGPLKGILGYETKPLVSTDYTNDTRSSII
DALSTQVIDKTMIKIYAWYDNEAGYSKRMAELCNIVAAMNITGQEPSFKYE

>prePGK_29157 plastid (pre-sequence) (JGI 29157) plastid

MKFVQAAIFALAASASTTAAFAPAKTFGVRSFAP

>PGK_51125 cytosol (193 Amino acids) (JGI 51125) cytosol

MASDMPKLAPGATRKRNVFDVIEALQKQSAKTILVRVDFNVPMNSDGKITDDSRIRGALPTIKAVVNAKCNAVLV
SHMGRPKLVQKAADDEETRQQRHELSLKPVADHLAKLLDQEVLFGDDCLHAQSTIRELPAEGGGVCLLENLRFYK
EEEKNGEDFRKTLASYADGYVNDAFGTSHRAHASVAGVPALLP

>PGM_43812 unclear (130 Amino acids) (JGI 43812) unclear localization, cytosol plus ER or mitochondria

MGRRTTHRRLFPALALIFAELIMSTAYSLAWRTSAACWTTTTGTACSRSRIATTRKVRRSRPNPCNPWHPVAFSF
FGTSSRRCRSSGSLYGEIDADAEGPDSPSADDRSVPTPSTTSSLSRSETLPPIPP

>PGM_43253 mitochondria (112 Amino acids) (JGI 43253) mitochondria

MASITLNRSRFTMITAIGMSHPRSHGTPRSVLLLLLRQFSSKDWNSKGTDSASRSGPVLIKKTPRSAAAAKLRST
APSLNGSTTDSTTGAVKHHPAHHYINGGTPCDPAPPP

>PGM_26201 unclear (408 Amino acids) (JGI 26201) unclear, mitochondria or ER

MLVPHPSGKAMRGLREEACRFLSSRSFGATLDATHARMGGNFVNSVQACNNGKRVCWHQRNRRTFSVVATQRNGI
GHRTTQGETEAVPRRHFTSLNQSTPFQLCFLRHGQSTWNRDNIFIGWTDTPLTDDGVLEARVAGKMLHKSGIRFD
EVHTSLLRRSIRTTNLALMELGQEYLPVHKHWRLNERCYGDLVGKNKKEVVMQHGADQVKRWRRSYDEPPPPMSD
DHPYHPARDPRYQNILDELPKSESLKNTVERSSLYWDEVLAPALREGKTLLVVGHENNLRSLLMRLEDIAPEDII
NLSLPRAVPLAYRLDENLKPLPREDGKLDEATGFLKGTWLGGDQAVSEILDRDHKQVYDTAITTNLEIGQDREKW
NNWMEFIMGKPSAKQKRIGGDKQNGFAGGAAIP

>PGM_42857 plastid (175 Amino acids) (JGI 42857) plastid

MAMDAITMRKLTLTMAVLLIVSGCEALLVFLPRRSPFTVISTRSSTNSAGLLHLHSKANESDGLEGKWIKVSSAL
DEGVDAANEEKEGAFLSSDYNSMNGYNTDLNRYHTMLRERGTFVEALFGQRRSFVIAKRDGDENEDGWRDMRRQR
RPLWKHLLRLPISVAKNVLWKPPQP

>PGM_35164 mitochondria (351 Amino acids) (JGI 35164) mitochondria

MRIPCRRLHPQLSAKGTRRPFQYSSSNSIDDQHRSSHLDASPGRHIVVRHGQSVWNKGSNQLERFTGWTNVGLSE
NGQRQAVQAARKLHGYSIDCAYVSLLQRSQATLRLMLEELNDQGRRSEGYDDLTTDIPVISSWRLNERHYGALTG
QSKLQAEQLFGKAQLDLWRYSYKIPPPPMDPDTFSSWKHQAHCQMATYIHHRHNRSRVIEKGNSVWDSSRAVMPR
SEAFFDVLQRIVPLWKYGIAPRLARGETVLLVGHANSVKALLCLLDPHTVTPTSIGALKIPNTTPLVYQLIRDYP
GASTSVPASFPVLGDLRVVIPPSNSTRYPLSGTWLEDPPVARDAGTAVEEP

>PGM_51298 cytosol (131 Amino acids) (JGI 51298) cytosol. If a shorter version, starting from the second Methionine is used, a localization at the plastid as a blob like structure is the result (data not shown).

MCDESRQTATPMIHFEIFRFSDPLVRQDRQAPHLSLTSTVKILSDSNLHKLFIMMLRSLVLALSWTVASAFTHQS
TFWGRTAVTNSRILSLSPPTDASSSALCMKYMLVLVRHGESTWNKENRFTGWVDCP

>preENO_56468 cytosol (66 Amino acids) (JGI56468) cytosol. Start Methionine from GFP was not included in the construct.

MLFKPSTLLALFAVAGTTLAFAPRSTTTPLTSTTRGSASSSVTTLAMSGITGVLAREILDSRGNPV

>ENO_56468 plastid (443 Amino acids) (JGI56468) plastid

MLFKPSTLLALFAVAGTTLAFAPRSTTTPLTSTTRGSASSSVTTLAMSGITGVLAREILDSRGNPTVEVEVTTAD
GVFRASVPSGASTDAYEAVELRDGGDRYMGKGVLQAVQNVNDILGPAVMGMDPVGQGSVDDVMLELDGTPNKANL
GANAILGVSLAVAKAGAAAKKVPLYRHFADLAGNNLDTYTMPVPCFNVINGGSHAGNKLAFQEYFVIPTGAKSFA
EAMQIGCEVYHTLGKIIKAKFGGDATLIGDEGGFAPPCDNREGCELIMEAISKAGYDGKCKIGLDVAASEFKVKG
KDEYDLDFKYDGDIVSGEELGNLYQSLAADFPIVTIEDPFDEDDWENWSKFTTKNGATFQVVGDDLTVTNIEKIE
RAIDEKACTCLLLKVNQIGSISESIAAVTKAKKAGWGVMTSHRSGETEDTYIADLAVGLCTGQIKTGA

>PK_49098 cytosol (507 Amino acids) (JGI: 49098) cytosol

MTASQTKITASGPELRGANITLDTIMKKTDVSTRQTKIVCTLGPACWEVEQLESLIDAGLSIARFNFSHGDHEGH
KACLDRLRQAADHKKKHVAVMLDTKGPEIRSGFFADGAKKISLVKGETIVLTSDYSFKGDKHKLACSYPVLAKSV
TPGQQILVADGSLVLTVLSCDEAAGEVSCRIENNAGIGERKNMNLPGVIVDLPTLTDKDIDDIQNWGIVNDIDFI
AASFVRKASDVHKIREVLGEKGKGIKIICKIENQEGMDNYDEILEATDAIMVARGDLGMEIPPEKVFLAQKMMIR

QANIAGKPVVTATQMLESMITNPRPTRAECSDVANAVLDGTDCVMLSGETANGEYPTAAVTIMSETCCEAEGAQN
TNMLYQAVRNSTLSQYGILSTSESIASSAAKTAIDVGAKAIIVCSESGMTATQVAKFRPGRPIHVLTHDVRVARQ
CSGYLRGASVEVISSMDQMDPAIDAYIERCKANGKAVAGDAFVVVTGTVAQRGVTNA

>PK_56445 cytosol (538 Amino acids) (JGI 56445) cytosol

MSLSQSSDVPILAGGFITLDTVKHPTNTINRRTKIVCTIGPACWNVDQLEILIESGMNVARFNFSHGDHAGHGAV
LERVRQAAQNKGRNIAILLDTKGPEIRTGFFANGASKIELVKGETIVLTSDYKFKGDQHKLACSYPALAQSVTQG
QQILVADGSLVLTVLQTDEAAGEVSCRIDNNASMGERKNMNLPGVKVDLPTFTEKDVDDIVNFGIKHKVDFIAAS
FVRKQSDVANLRQLLAENGGQQIKICCKIENQEGLENYDEILQATDSIMVARGDLGMEIPPAKVFLAQKMMIREA
NIAGKPVITATQMLESMINNPRPTRAECSDVANAVLDGTDCVMLSGETANGPYFEEAVKVMARTCCEAENSRNYN
SLYSAVRSSVMAKYGSVPPEESLASSAVKTAIDVNARLILVLSESGMTAGYVSKFRPERAIVCLTPSDAVARQTG
GILKGVHSYVVDNLDNTEELIAETGVEAVKAGIASVGDLMVVVSGTLYGIGKNNQVRVSVIEAPEGTVKETPAAM
KRLVSFVYAADEI

>PK_45997 cytosol (533 Amino acids) (JGI 45997) cytosol

MLSSTSTIPKLDGEVVTLSIIKKPTETKKRRTKIICTLGPACWSEEGLGQLMDAGMNVARFNFSHGDHEGHGKVL
ERLRKVAKEKKRNIAVLLDTKGPEIRTGFFADGIDKINLSKGDTIVLTTDYDFKGDSKRLACSYPTLAKSVTQGQ
AILIADGSLVLTVLSIDTANNEVQCRVENNASIGERKNMNLPGVVVDLPTFTERDVNDIVNFGIKSKVDFIAASF
VRKGSDVTNLRKLLADNGGPQIKIICKIENQEGLENYGDILEHTDAIMVARGDLGMEIPSSKVFLAQKYMIREAN
VAGKPVVTATQMLESMVTNPRPTRAECSDVANAVYDGTDAVMLSGETANGPHFEKAVLVMARTCCEAESSRNYNL
LFQSVRNSIVIARGGLSTGESMASSAVKSALDIEAKLIVVMSETGKMGNYVAKFRPGLSVLCMTPNETAARQASG
LLLGMHTVVVDSLEKSEELVEELNYELVQSNFLKPGDKMVVIAGRMAGMKEQLRIVTLDEGKSYGHIVSGTSFFF
ERTRLLDF

>PK_56172 mitochondria (86 Amino acids) (JGI: 56172) mitochondria

MFRRAVLSLSTRAIRTPVPCSVARGDASQVRSLAQTTFYLPDPADRSQDVHNRGNLQLSKIVATIGPTSEQEEPL
RLVTDAGMRIM
